# Development and Assessment of a Diagnostic DNA Oligonucleotide Microarray for Detection and Typing of Meningitis-Associated Bacterial Species

**DOI:** 10.3390/ht7040032

**Published:** 2018-10-16

**Authors:** Stephanie A. Bannister, Stephen P. Kidd, Elizabeth Kirby, Sonal Shah, Anvy Thomas, Richard Vipond, Michael J. Elmore, Andrew Telfer Brunton, Peter Marsh, Steve Green, Nigel J. Silman, Karen E. Kempsell

**Affiliations:** 1 Public Health England, Porton Down, Salisbury SP4 0JG, UK; stephanie_vollmer@hotmail.com (S.A.B); Stephen.Kidd@hhft.nhs.uk (S.P.K.); lizkirby2001@yahoo.co.uk (E.K.); sonal.shah@lshtm.ac.uk (S.S.); Anvy.Thomas@phe.gov.uk (A.T.); Richard.Vipond@phe.gov.uk (R.V.); Mike.Elmore@phe.gov.uk (M.J.E.); Nigel.Silman@phe.gov.uk (N.J.S.); 2 Department of Clinical Microbiology, Royal Cornwall Hospitals NHS Trust, Penventinnie Lane, Treliske, Truro, Cornwall TR1 3LQ, UK; latbex2@hotmail.com; 3 Public Health England Laboratory Southampton, Southampton General Hospital, Tremona Road, Southampton SO16 6YD, UK; Peter.Marsh@phe.gov.uk (P.M.); Steve.Green@phe.gov.uk (S.G.)

**Keywords:** meningitis, microarray, bacterial, diagnostic, assay, ArrayTube, diagnosis, infection

## Abstract

Meningitis is commonly caused by infection with a variety of bacterial or viral pathogens. Acute bacterial meningitis (ABM) can cause severe disease, which can progress rapidly to a critical life-threatening condition. Rapid diagnosis of ABM is critical, as this is most commonly associated with severe sequelae with associated high mortality and morbidity rates compared to viral meningitis, which is less severe and self-limiting. We have designed a microarray for detection and diagnosis of ABM. This has been validated using randomly amplified DNA targets (RADT), comparing buffers with or without formamide, in glass slide format or on the Alere ArrayTube^TM^ (Alere Technologies GmbH) microarray platform. Pathogen-specific signals were observed using purified bacterial nucleic acids and to a lesser extent using patient cerebral spinal fluid (CSF) samples, with some technical issues observed using RADT and glass slides. Repurposing the array onto the Alere ArrayTube^TM^ platform and using a targeted amplification system increased specific and reduced nonspecific hybridization signals using both pathogen nucleic and patient CSF DNA targets, better revealing pathogen-specific signals although sensitivity was still reduced in the latter. This diagnostic microarray is useful as a laboratory diagnostic tool for species and strain designation for ABM, rather than for primary diagnosis.

## 1. Introduction

Meningitis is a disease caused by inflammation of the meninges, the cause of which is generally associated with infection [1,2,3]. It can present acutely or chronically with less severe symptoms over a prolonged period. Acute meningitis is a syndrome defined by meningeal symptoms (headache, neck stiffness, vomiting, and photophobia) in conjunction with cerebral dysfunction (confusion, coma) and progresses over hours to days. The cerebral spinal fluid (CSF) often presents with leukocytosis. The most common causes of acute meningitis in the United Kingdom are bacterial and viral infection. 

Viral infections are responsible for aseptic meningitis syndrome, which is characterized by a lymphocytic pleocytosis in the CSF and negative bacterial cultures. However, this form of meningitis can be mimicked by intracellular bacterial infections including *Rickettsia* spp., *Leptospira* spp. and *Mycobacterium tuberculosis*. Acute viral meningitis (AVM) tends to occur in epidemics and is usually a mild, self-limiting disease in the immunocompetent patient. It can be caused by a wide range of viral agents, but typically enteroviruses and herpesviruses [4,5,6]. Despite the usually mild nature of these viral infections, they can also be associated with more serious acute viral encephalitis (AVE), which has high mortality rates and survivors can develop chronic conditions, including significant neurological deficits.

Acute bacterial meningitis (ABM) can cause more fulminant disease [3,7,8] and progression to a critical life-threatening septicemia can be extremely rapid. This has high mortality and morbidity rates if treatment is delayed or withheld [9,10,11]. The causes of ABM vary and are dependent on the immune status and age of the patient and whether any recent head surgery or trauma has preceded symptoms. The most common causes in healthy adults are *Haemophilus influenzae*, *Streptococcus pneumoniae (*pneumococcal disease*)* and *Neisseria meningitidis* (meningococcal disease). In neonates, the elderly and immunocompromised individuals *Escherichia coli*, *Streptococcus agalactiae* (GBS) *Listeria monocytogenes* and *Mycobacterium tuberculosis* are the prevalent pathogenic causes. *Staphylococcus aureus*, *Staphylococcus epidermidis*, and *Cutibacterium acnes* can be the causative agents in postcranial surgery and CSF shunts and trauma and need to be excluded during primary diagnosis [12,13].

In the early stages of disease the etiological agents responsible for ABM, AVM, and AVE are extremely difficult to diagnose due to clinical symptoms of a diffuse and nonspecific nature [14,15,16]. Therefore, empirical treatments of Ceftriaxone and Acyclovir, plus or minus Dexamethasone or Amoxicillin are administered a soon as possible and pre-emptive isolation of the patient is undertaken until a diagnosis is made [17]. Currently, a combination of predictive factors are used to form an initial differential diagnosis, including clinical presentation, CSF cell count and biochemistry investigations [18]. This is then followed by CSF viral PCR and bacterial culture. Serology and imaging of the brain may help to confirm a diagnosis and inform treatment. However, some causative agents e.g., *N. meningitidis*, can be difficult to recover using culture-based methods, in part due to the usual practice of administering antibiotics prior to patient sample collection [19,20]. Many diagnostic laboratories therefore augment their diagnostic capabilities with the use of molecular methods for identification of meningitis-associated bacterial [21,22] and viral pathogens [23,24,25,26], although implementation of these is not comprehensive. These are primarily real-time qualitative Polymerase chain reaction assays (RT-PCR), which are available for a limited number of meningitis-associated pathogens. In addition, there are some molecular tests available to discriminate between different bacterial species or serogroups; e.g., *N. meningitidis* serogroups B, C, Y and W135 [27,28,29,30], *S. pneumoniae* [11] and *H. influenzae* [31]. Serogrouping of most meningitis-associated strains is conducted using a range of methods e.g., culture, serology and immunological tests at reference laboratories [14] and more latterly sequencing [32], however there is still a need for harmonization of methodologies between laboratories [33].

There is a need for development of other rapid and sensitive diagnostic methods with the capability for simultaneously assaying for multiple bacterial and viral agents and which discriminate between different strains and serogroups. To facilitate this development we have investigated the use of microarrays as a solution for rapid molecular pathogen identification of ABM. These have been the focus of considerable attention in previous years for a range of analytical processes [34,35,36]. The use of these multiplexed analysis systems has been widely accepted previously, as a means for augmenting frontline traditional diagnostics, as they offer many advantages over traditional monoplex molecular technologies. Microarrays are also highly amenable to multiplexing, obviating the need for multiple tests per patient [37,38], with the potential for saving time and cost. Microarrays have further benefits over established block-based or real-time PCR tests because they require less prior knowledge of the infectious agent [39,40,41,42].

Diagnostic microarrays have previously been developed for detection of bacteria and viruses from both environmental and clinical sources. A number of groups developed low-complexity diagnostic microarrays for identification of microorganisms in water and food samples [43,44,45]. They have also been developed for detection of pathogens in clinical material based on genus and/or species-specific oligonucleotide probes designed from database gene sequence alignments, including viral [46,47,48,49], bacterial [50,51,52,53], fungal [54] and eukaryotic pathogens [55,56]. Wang and De Risi and colleagues developed a number of arrays for detection of known viral and emerging viral pathogens using hybridization to species-specific oligonucleotide probes [57,58,59] and using advanced bioinformatics analysis tools to reveal pathogen-specific signal patterns [60]. Similar methodologies have been used by these and other groups to identify meningitis-associated and emerging pathogens, including the severe acute respiratory syndrome (SARS) coronavirus [61,62,63,64]. Here we present development and validation of a meningitis diagnostic microarray; it’s reconfiguration onto the Alere Arraytube^TM^ (Alere Technologies GmbH, Jena, Germany) platform and provide recommendations for its use in a diagnostic setting.

## 2. Materials and Methods

### 2.1. Culture of Bacterial Pathogen Strains and Nucleic Acid Purification

All bacterial species and strains used in this study are shown in Supplementary Material S1, Table S1. *N. meningitidis* strains representing serogroups A, B, C and W135, *N. flavescens* and *N. lactamica* strains 020 & Y92-1009 were sourced from Prof. A. Gorringe, Meningitis Research Group, PHE, Porton Down, UK. All other *N. meningitidis* strains were obtained from the National Collection of Type Cultures, Colindale, London UK. Genomic DNA from all Neisseria strains were purified as follows. Bacteria were cultured overnight on 5% horse blood/brain heart infusion agar plates at 37 °C; colonies were isolated and resuspended in 200 μL Tris EDTA Glucose (TEG) buffer (10 mM Tris-Cl pH 8.0, 1 mM EDTA, 2% glucose), then 500 μL guanidine hydrochloride lysis buffer (8 M guanidine hydrochloride, 0.2% sodium sarkinosate) was added to the bacterial suspension which was then mixed by inversion. Two-hundred microliters of chloroform were then added to the emulsion which was centrifuged at 13,000× *g* for 10 min. The DNA was recovered from the upper aqueous layer by precipitation with 100% ethanol and collected by centrifugation. After washing twice with 70% ethanol the DNA pellet was air dried and re-suspended in TE buffer (10 mM Tris-Cl pH 8.0, 1 mM EDTA), then stored at −20 °C prior to use. Purified nucleic acid samples from other nonmeningococcal bacteria were obtained from coworkers at Public Health England, Southampton, UK (PHES). 

### 2.2. Collection of Human Donor Blood and Patient Cerebrospinal Fluid Samples and Purification of Nucleic Acids

Seven CSF samples were collected initially at Public Helath England (PHE) for routine testing for meningitis pathogens. Retained clinical samples acquired for diagnostic purposes are a valuable resource and PHE has dispensation to use anonymised patient material for performance assessment, this lies outside the Human Tissue Act 2004 (according to RCPath guidelines G035 [65]. These were processed for DNA nucleic acids as pellet and supernatant fractions using a modification of the QIAamp DNA Mini Purification Kit (Qiagen, Skelton House, Manchester, UK), as follows. CSF samples were centrifuged for 5 min at 1000× *g* to remove cellular debris, 200 μL aliquots of the supernatant fraction were then removed and centrifuged further at 13,000× *g* for 10 min. The pellet and supernatant fractions were recovered and processed separately. The pellet fraction was resuspended in 200 μL of TE buffer prior to further processing. Nucleic acids from each pellet and supernatant fraction were then purified using the Qiagen QIAamp DNA Mini Kit according to the manufacturer’s instructions recovered nucleic acids were stored frozen at −20 °C until required. Human AB whole blood was purchased from Sigma (Sigma-Aldrich, Dorset, UK) and whole blood DNA purified using the QIAamp DNA blood mini kit (Qiagen), according to the manufacturer’s instructions. 

An additional 146 CSF samples were collected by lumbar puncture for suspected bacterial or viral meningitis/encephalitis between March and November 2011 at PHES. The volumes of CSF obtained differed between patient samples, therefore phosphate buffered saline (PBS; Sigma, Dorset, UK) was added to low volume samples below the minimum extraction volume, to make a final total volume of 200 μL. Nucleic acids were then purified using the QiaSymphony automated DNA extraction platform and the QiaSymphony DSP virus/pathogen mini-kit, according to the manufacturer’s instructions (Qiagen). All pathogen, human blood and patient DNA samples were quantified by spectrophotometric analysis at 260 nm prior to use.

### 2.3. Design of Oligonucleotide Microarray Probes

The oligonucleotide probes used in this array were a mixture of 50 and 70-mers (all sequences are given in Supplementary Material S2, Tables S2 and S3). Genus-specific probes were designed from alignments of 16S rRNA sequences as described previously [66]. All other oligonucleotide probes e.g., for the sialyl-transferase genes of *N. meningitidis*, were designed from alignments of bacterial species or strain-specific gene sequences using the Clustal W function [67] of either Megalign [68] or Bioedit [69]. Seventy-mer regions of low sequence conservation were selected, which differed significantly from other closely related species or strains and which were discriminatory for the requisite pathogen. The species or strain specificity of these probes was confirmed using database search algorithms e.g., BLAST [70]. Oligonucleotides probes with greater than 90% sequence identity with other closely-related bacterial gene sequences were excluded. The relative melting temperatures (Tm) of each probe was calculated and those with a Tm of 60 ± 8 °C were selected. The suitability of the selected oligonucleotides for their use as discriminatory probes with respect to low self-complementarity and hairpin loop formation was also assessed using the algorithm Oligonucleotide Calculator), prior to inclusion on the array. 

### 2.4. Printing of Microarray Oligonucleotide Probes in Glass Slide Format

All 50- and 70-mer bacterial species and strain oligonucleotide probes for the glass slide format microarray were synthesised by Illumina (Illumina, Cambridge, UK) and printed in quadruplicate onto epoxy-coated Nexterion E slides (Schott Ltd., Stafford, UK) using a BioRobotics Microgrid II gridder (Digilab Inc., Hopkinton, MA, USA). Oligonucleotides were diluted to a final concentration of 20 μM in 1.5× saline-sodium citrate buffer ((SSC) 25% dimethyl sulfoxide and 0.005% (*w*/*v*) sodium dodecyl sulphate) prior to printing. The slides were then air-dried, baked at 80 °C for 2 h and stored with desiccant at ambient temperature in the dark prior to use.

### 2.5. Amplification and Cy3-Labelling of Purified Nucleic Acids and Hybridization to the Meningitis Glass Slide Array

All hybridizations in this study were conducted using Cy3-labelled DNA targets from either purified pathogen genomic, human blood or patient CSF DNAs. The method used to generate randomly amplified DNA targets is a modification of a previously published method [71] and has been described previously [72]. 

#### 2.5.1. Hybridization to Pan-Pathogen Arrays Using Randomly-Amplified Cy3 Labelled Targets and the Manual Hybridization Method 

Glass slide format microarray slides were hybridized with amplified, Cy3-labelled target DNAs as published previously [53], with minor modifications. Slides were prehybridized in 5× SSC, 0.1% SDS and 4× Denhardts solution (Sigma-Aldrich), washed in sterile, nuclease-free water, followed by 100% isopropyl alcohol then air-dried. Cy3-labelled randomly-amplified target DNAs were denatured at 95 °C for 3 min then diluted to a final concentration of 80 μg/mL in 5× SSC buffer (Sigma-Aldrich), 0.1% SDS, 4× Denhardts solution. Forty microlitres of this hybridization mix were then applied to the microarray slide, covered with a glass coverslip, placed in a humidified multislide chamber (Genetix Inc., Wallsend, UK), then incubated at 50 °C for 16 h. The slides were then washed once in each of the following buffers for two minutes: (A) 1× SSC, 0.2% SDS, 50 °C, (B) 0.1× SSC, 0.2% SDS, 50 °C, (C) 0.1× SSC, 0.2% SDS, 20 °C, then centrifuged to dryness at 213× *g* for 5 min then scanned using an Affymetrix 428 microarray laser scanner at a gain of sixty.

#### 2.5.2. Hybridization to Pan-Pathogen Arrays Using Randomly-Amplified Cy3 Labelled Targets Using the Advalytix Slidebooster™ Hybridization Station.

Nonspecific hybridization to unrelated oligonucleotides was observed to a number of oligonucleotide probes on the array, using both pathogen and nonpathogen labelled nucleic acids. To address this issue and to attempt to reduce these events, nonmanual hybridizations were conducted using a Advalytix Slidebooster™ hybridization (Beckman Coulter UK, High Wycombe, UK) and wash station with addition of a blocking agent i.e., Denhardts solution and 16% formamide to the hybridization buffer to reduce background from nonspecific hybridization [73].

Microarray slides were prehybridized as described for manual hybridizations then placed array side up into a Slidebooster hybridization chamber containing sonic agitation chips, with coupling fluid placed between the slide-base and the platform, according to the manufacturer’s instructions. Lifter-slips were then placed on the slide, gasket side down and the slide left to preheat to 50 °C, prior to addition of labelled target. Preprepared target DNAs (Cy3-labelled randomly-amplified targets DNAs denatured at 95 °C for 3 min, then diluted to a final concentration of 120 μg/mL in 5× SSC, 0.1% SDS, 4× Denhardts solution, 16% formamide at 50 °C) were then applied to the Lifter-slip edge, drawing the labelled target onto the array by capillary motion. Use of this platform was found also to significantly reduce the time of incubation needed. Thus, the slides were then incubated at 50 °C for six hours, at a maximum mixing power of 27. After incubation the slides were removed, placed in an Advawash™ chamber and washed according to the protocol for manual hybridizations described above. The slides were centrifuged to dryness at 213× *g* for 5 min and then scanned using an Affymetrix 428 microarray laser scanner at a gain of sixty.

### 2.6. Data Processing and Analysis

For glass slide array hybridizations, files were saved in Tiff format after image capture, then quantified using the scan analysis software Bluefuse™ (BlueGnome Inc., Cambridge, UK), all data were normalized to the global median for each slide then replicate data points fused according to median fluorescent intensity and identity. The data was log-transformed log_2_() function in “R”. [74]. To identify probes with significantly higher Fluorescence a Z-score analysis was undertaken [75]. The Z-score is the number of standard deviations a given spot intensity is away from the mean, and therefore a measure of which signal(s) differ significantly from the expected value.

$$Z_{i}\;=\;\frac{S_{i}\;-\;\mu }{\delta }$$

where *Z_i_* is the Z-score for the *i*th spot, S*_i_* is the fluorescent intensity of the *i*th spot, μ and δ are the mean and standard deviation of all sample spot intensities, Z-scores are then converted into probabilities. In this study probability values were calculated directly from the intensity data using the pnorm() function of ‘R’ base package [74]

*p* <−1 − pnorm (S*_i_*, μ, δ) (1)

Probes were sorted lowest to highest *p*-value and those with less than a user-defined cut-off (in this case ≤0.2) were considered positive. Pathogen species and serogroups assignations were given to each test target based on the number of significant generic, species-specific and/or serogroup-positive probes in any given ranked data set. Fluorescence intensities for all raw data points were depicted pictorially in heatmap format using an algorithm written in the statistical package ‘R’, published previously [74,76].

### 2.7. Reconfiguration of the Pan-Pathogen Array in Alere ArrayTube™ Format

Microarray hybridizations in glass slide format are not readily amenable for use as a routine diagnostic assay, as there are limitations in terms of assay set up, ease of use and scale-up for high-throughput. We therefore decided to transition our best performing validated detection probes to an alternative assay format which would be more amenable to routine diagnostic laboratory use. A selection of our preferred best performing meningitis-associated bacterial probes were chosen for transition onto the ArrayTube^TM^ system, using information on probe performance ascertained from prior validation of the array in glass slide format. A more focused bacterial meningitis array probe set was constructed for evaluation of this form set, which included probes for *N. meningitidis* strains A, B, C, X, Y, W135 and L, *H. influenzae*, *S. agalactiae*, *S. pneumoniae*, *S. aureus* and *L. monocytogenes* plus additional test and control probes (for pathogens *Mycobacterium tuberculosis*, *Escherichia coli* K12, Human G6PD1, PGK1 and B2M1 and *Arabidopsis thaliana* control sequences (Supplementary Material S2, Table S3). All DNA probe sequences were synthesised and printed in ArrayTube^TM^ format by Alere Technologies GmbH, Jena, Germany.

### 2.8. Development of a Targeted DNA Amplification and Labelling System Using VisualOMP Primer Design Software 

Primers for a targeted amplification system were designed in VisualOMP (DNASoftware, ANN Arbor, MI, USA), using a database of imported probe and gene target sequences. The software provides a means for in silico modelling of multiplex amplification steps. This modelling process provides a solution for rational design of comprehensive multiplex specific primer sets, with similar physiochemical parameters using uploaded gene sequences as a reference. All gene sequences from which the microarray probes had been designed (excluding *bexA*) and those for newly designed test and control probes were uploaded into the program and modelled using default settings. Final select primer pairs were again assessed for specificity by comparison with database sequences using the BLAST algorithm, before use [77]. These are given in Supplementary Material S3, Table S4. 

### 2.9. Amplification and Labelling of Target DNAs and ArrayTube Hybridizations

All HPLC purified nucleotide primers were synthesized by Eurofins MWG Operon (Ebersberg, Germany). A targeted, noncompetitive amplification system, based on the previously published protocol of Bolander and coworkers [72], was used for amplification and labelling of target nucleic acids in this study. Amplification and biotin labelling of all pathogen, control and human DNAs were performed using Phusion Taq polymerase from Finnzymes (New England Biolabs, Hitchin UK), using the following amplification protocol; 10.0 µL Phusion Taq buffer (5×), 125 μM dNTPs (excluding dUTP), 75 μM dUTP and 75 μM Biotin-16–dUTP (New England Biolabs, USA), 0.5 µL Phusion Taq, Primer Mix (0.5 μM each Primer final concentration), DNA Template 5.0 µL in a final volume of 50 μL. PCR amplification protocol—one cycle of 98 °C for 120 s, followed by 35 cycles of 98 °C for 15 s, 63 °C for 30 s, 72 °C for 45 s, with a final step of 72 °C for 420 s. Labelled target hybridizations were conducted in printed ArrayTubes^TM^ using the Alere hybridization protocol, according to the manufacturer’s instructions (Alere Technologies GmbH) and using Poly-HRP Streptavidin (Fisher Scientific-UK Ltd., Loughborough UK), with one minor alteration, using a final hybridization volume of 200 µL. ArrayTube^TM^ incubations were performed on a Thermomixer Comfort Microtube shaking heater block (Eppendorf UK Limited, Stevenage UK) and were read immediately after incubation with substrate using an ATR03 ArrayTube^TM^ analyser. Hybridization signal intensities, data export and analyses were conducted using Iconoclust ArrayTube^TM^ software according to the manufacturer’s instructions (Alere Technologies Gmb). Mean intensities from each probe spotted in duplicate were used in the analysis, probes with mean values ≥0.4 were considered positive, based on value cut-offs established by Batchelor and colleagues [78].

### 2.10. Real-Time Qualitative PCR Assays 

To confirm the results obtained for pathogen target ArrayTube^TM^ hybridizations, pathogen-specific RT-PCR assays were used. Multiplex meningitis-associated pathogen real-time PCR assays were constructed based on either previously published assays for many of the bacterial pathogens represented on the array e.g., Group B Streptococcus [79], *H. influenzae* [21,80], *S. aureus* [81], *S. epidermidis* [2], *N. meningitidis* [14,82] and *S. pneumoniae* [83], or in house designed PHE assays, where existing previously published assays were not available. Real-time PCR assays to the meningococcal capsular transport gene *ctrA* were used for *N. meningitidis*, as previously described [21]. These assays had previously all been run in singleplex, however for ease of use in assaying large numbers of samples these were reconfigured to run as three duplex assays (Supplementary Material S4, Table S5). An additional assay for *L. monocytogenes* was sourced [84] and run in single-plex. 

Real-time PCR primers and probes were synthesised by Eurofins MWG Operon (Germany). A Taqman Universal Mastermix kit (Applied Biosystems Inc., Foster City CA USA) was used per the manufacturer’s instructions and all assays were run on ABI 7500 Thermocyclers (Applied Biosystems Inc., Foster City CA USA) All assays were validated using purified, species-specific pathogen nucleic acids, prior to use on clinical samples. For the real-time PCR reaction, primers were used at a concentration of 300 nM, and the internal, labelled probe at 200 nM, with 12.5 µL of TaqMan Universal PCR master mix (Applied Biosystems Inc., Foster City CA USA). Five microlitres of sample were added to generate a total reaction volume of 25 µL. Real-time PCRs were performed in 96-well reaction plates as follows: 2 min at 50 °C, 10 min at 95 °C, 50 cycles, of 95 °C for 15 s and 60 °C for 60 s, probe fluorescence was detected in the 530 nm channel, the assay was completed at 50 cycles.

## 3. Results

### 3.1. Hybridization of Randomly Amplified DNA Targets to the Meningitis Pan-Pathogen Glass Slide Printed Array

**Figure 1:**
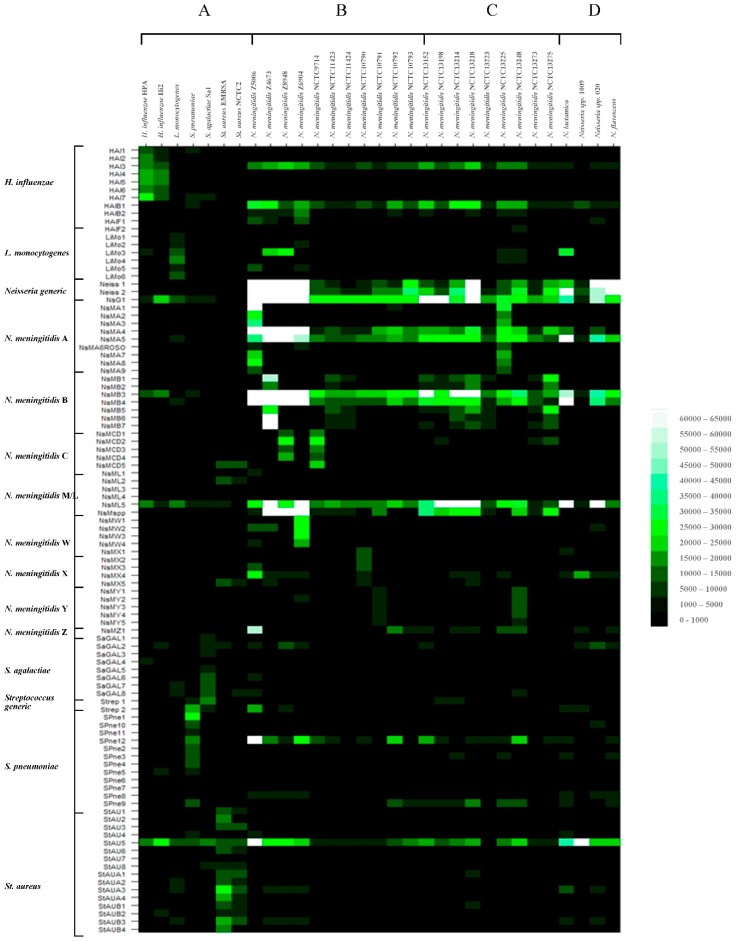
Fluorescence intensity of probe hybridization with amplified Cy3-labelled bacterial DNA targets using purified pathogen nucleic acids to the glass slide-printed meningitis microarray, depicted in heatmap format (**A**) nonmeningococcal pathogens, (**B**) typed meningococcal pathogens, (**C**) untyped meningococcal pathogens, and (**D**) nonmeningococcal *Neisseria* spp.

All meningitis-associated bacterial pathogen and control human donor blood DNA samples were randomly amplified, labelled with Cy3-dCTP and hybridized to the pan-pathogen array. The results using randomly-amplified bacterial DNA targets hybridized to the meningitis array in glass slide format using the manual hybridization methods and formamide-free buffers are depicted in heatmap format in Figure 1. Exported data values were normalized to the global median, divided by the human DNA control value for each probe, then significance values calculated for each probe set, using the *t*-test function in Excel (one-tailed distribution, paired). Probes were ranked (highest to lowest intensity), according to these corrected data values and using the data sort function in Excel, from which species-specific probe hybridization profiles were discerned. Ranked data values for each pathogen or control DNA and their associated significance values are depicted graphically in Supplementary Materials S5 and S6. Most amplified bacterial genomic targets showed good specific binding to their relevant species-specific probes. However some rogue nonspecific binding of bacterial DNAs to unrelated probes was also observed, particularly with HAI3, HAIB1, NsG1, NsMB3, MsML5, SPneu12 and StAU5. Although great care was taken in design to optimize oligonucleotide probe with regards to secondary structure, specificity and melting temperature etc., these events may still be due to residual secondary structure [85].

#### 3.1.1. Hybridization Profiling of Nonmeningococcal Bacterial Pathogens

Hybridizations to the meningitis array using *H. influenzae* strains HPA and Hi2, *L. monocytogenes* strain Lm1, *S. agalactiae* strain Sa1, *S. pneumoniae* strain NCTC 07465 and *S. aureus* strains EMRSA-16 and ATCC 29213 generated pathogen-specific hybridization profiles as expected (Figure 1A and Supplementary Material S5), with some cross-hybridization to unrelated probes, as seen with all samples including the human control DNA. Although the pattern of cross- hybridization with most rogue probes (outlined above) was consistent, there was some minor variation in cross-hybridization to nonspecific probes between different pathogen DNA targets.

DNA targets amplified from *H. influenzae* strains HPA and Hi2 gave good hybridization intensities for seven of eleven of their species-specific probes. All probes for nucA and cpdB exhibited good species specificity, but not those for *bexA* suggesting these latter gene probes are not useful as diagnostic targets for *H. influenzae* in this study. These strains may perhaps be capsule deficient [86]. The amplified DNA target from *L. monocytogenes* strain Lm1 showed good binding to all its pathogen-specific probes. The DNA target from *S. pneumoniae* NCTC07465 bound well to the *Streptococcus* 16S rRNA generic probes and nine of twelve of its specific probes. The DNA target from *S. agalactiae* strain Sa1 bound well to the *Streptococcus* 16S rRNA generic probes and seven of eight of the specific probes. DNA targets from *S. aureus* MRSA strain EMRSA-16 and MSSA strain ATCC29213 exhibited good binding to all but two of the sixteen *S. aureus* specific probes (however, as described above StAU5 exhibited a high degree of nonspecific binding to all bacterial DNA targets). Both *S. aureus* strains exhibited significant binding to the *N. meningitidis* serogroup-specific probe NsMCD5. Nonfunctional probes from *S. pneumoniae*, *S. agalactiae* and *S. aureus* did not appear to be specific to one gene as observed with *H. influenzae*. Thus, certain probes from these nonmeningococcal meningitis-associated pathogens exhibited target-specific hybridization profiles and show potential as diagnostic markers for the relevant organism. Other probes exhibited minimal binding or cross-hybridization with unrelated DNA targets and are not useful for future diagnostic development.

#### 3.1.2. Hybridization Profiling of Meningococcal Bacterial Pathogens of Known Serogroup

Good hybridization fluorescence intensities were observed to *N. meningitidis*-specific oligonucleotide probes with most *Neisseria* spp. amplified targets (Figure 1B, Supplementary Material S5). The 16S rRNA probes Neiss 1 and Neiss 2 and ubi gene probes NsMA5 and NsMB4 gave hybridization signals with all *Neisseria* spp. These probes, therefore, act as generic *Neisseria* probes. The type IV pilin gene probe NsMA4, hybridized with most *Neisseria* spp. targets. The pilin gene probes NsMB3 and NsG1 and the lcbA gene probe MsML5 showed nonspecific binding to other nonmeningococcal DNA targets. NsMspp showed binding with the majority of *N. meningitidis* strain DNAs with the exception of serogroups A, X, Z and Z-prime and the nonmeningococcal *Neisseria* spp.

Hybridization specificity to serogroup-specific probes generally corresponded well with the serogroup of the relevant strain. *N. meningitidis* serogroup A strain Z5005 DNA target bound to *Neisseria* generic probes and to all of the serogroup A probes (some of which exhibit some lack of specificity, as discussed previously e.g., NsMA4 and NsMA5). It also bound to some other serogroup probes, NsMZ1 and NsMW2. Thus, six of nine probes specifically designed for serogroup A exhibited group serospecificity, all designed to the sacB gene. The remaining probes exhibited some cross-reactive binding to other *Neisseria* spp. but not other non-Neisserial species bacterial DNA targets. Strains NCTC10792 and NCTC10793, designated Z and Z-prime respectively, showed binding to NsMZ1; NCTC10792 also bound to NsMA1. The Z serogroup-specific probe NsMZ1 does show binding with other *N. meningitidis* strain targets e.g., serogroup A strains, however these also show binding to their respective serogroup A-specific probes and are therefore easily distinguishable from Z strains. Z strains show binding to NsMZ1 only (with some weak additional binding to NsMA1 with strain NCTC10792). *N. meningitidis* strain Z4673 bound to the generic probes and all serogroup B-specific probes, as did strains NCTC11423 and NCTC11424 (previously annotated as strains 73-M6 and 74-M24) and not to serogroup A specific probes. These latter strains have therefore been previously incorrectly characterized. Only probes NsMB1 and NsMB2 and NsMB5-NsMB7 are in fact specific for serogroup B.

*N. meningitidis* serogroup C and D strains Z8948 and NCTC9714 respectively showed binding to the *Neisseria* generic probes and four of the five serogroup C/D probes. One of these latter probes showed nonspecific binding with other meningitis-associated bacterial targets; NsMCD5 exhibited binding with both NCTC9714 but also both *S. aureus* strain DNA targets. Thus, probes NsMCD1-NsMCD4 are diagnostic for *N. meningitidis* serogroup C or D strains. Serogroup W135, X and Y strains Z6904, NCTC10790 and NCTC10791 respectively, showed binding to both the generic and their appropriate serogroup-specific probes; in addition, NCTC10790 bound to probe NsMZ1. The human DNA control showed a high degree of cross-reactive binding to serogroup W probes NsMW1, NsMW3 and NsMW4. Therefore, may not appear useful as significant identifying probes for W strains, if used for patient clinical samples directly, but may be useful for typing of cultured meningococcal strains. The majority of *N. meningitidis* strains exhibited a characteristic binding profile indicative of their serogroup, which may be useful for diagnostic identification.

#### 3.1.3. Hybridization Profiling of Meningococcal Bacterial Pathogens of Unknown Serogroup 

Nine of eleven *N. meningitidis* strains of known serogroup demonstrated distinct binding profiles to generic and serogroup-specific probes. We then went on to assess a further nine *N. meningitidis* strains of previously unidentified serogroup with a view to assessing the potential of these probes for serogroup identification (Figure 1C, Supplementary Material S5). In addition to binding to Neisseria generic probes, *N. meningitidis* strains NCTC13152, NCTC13198, NCTC13214, NCTC13218, NCTC13223, NCTC13273 and NCTC 13275 showed patterns of binding indicative of B serogroup strains. Although strains NCTC13152, NCTC13198, NCTC13214, NCTC13273 and NCTC13275 showed some limited binding to other serogroup-specific probes, the dominant profile was serogroup B. Strain NCTC13225 showed the binding profile of serogroup A and strain NCTC13248 showed the binding profile of serogroup Y. All previously un-serogrouped strains tested could be ascribed to a serogroup by microarray hybridization profiling, however the capsule synthetic characteristics of these strains is not known. Patterns of binding were not identical to previously tested serogroup strains, suggesting some strain-specific gene sequence variation. 

#### 3.1.4. Hybridization Profiling of Nonmeningococcal *Neisseria* spp.

To ascertain the binding profile of nonmeningococcal *Neisseria* strains, amplified DNA targets from three *N. lactamica* and one *N. flavescens* isolates were amplified, labeled, and hybridized to the pan-pathogen array (Figure 1D, Supplementary Material S5). Clear binding to generic probes Neiss 2, Neiss 1, NsMA5 and NsMB4 was observed with the *N. lactamica* and *N. flavescens* isolates. *N. lactamica* strains HPA1 and 2006 also bound to NsMB3, NsG1 and NsML5, which also show cross-reaction with other non-*Neisseria* spp. targets. *N. lactamica* HPA1 and 2009 bound NsMA4, *N. lactamica* HPA1 alone bound to NsMX1. Therefore, in addition to expected binding to the 16S rRNA *Neisseria*-generic probes Neiss1 and Neiss2, these nonpathogenic isolates bound to other *Neisseria* generic probes; however, none bound to NsMA1, NsMspp or NsMZ1 (designed to the *ctrA* gene). These *Neisseria* spp. do appear to share some genetic characteristics in common with pathogenic *Neisseria* in that they appear to carry type IV pilin-like and ubiA gene loci.

### 3.2. Purification of DNAs from Patient CSF, Labelling and Array Hybridizations 

We established that pathogen and serogroup-specific hybridization profiles could be identified using our pan-pathogen array probe set, using highly pure pathogen DNAs as template. We therefore sought to test the method on culture negative CSF samples from patients with suspected meningitis, to establish whether pathogen-specific profiles could be identified. Nucleic acids were purified from patient CSFs as described above. The quantities of total nucleic acid recovered and sample nomenclature designations from these select patient CSF samples, are given in Supplementary Material S8, Table S14. As can be seen small amounts of nucleic acids were recovered from patient CSF samples after slow centrifugation to remove large cellular debris. Typically, there was a greater amount of DNA in the supernatant than the pellet fractions (ranging between 0–750 ng/uL total nucleic acids). 

#### 3.2.1. Hybridization Profiling of Patient CSF Target DNAs and Putative Pathogen Identification to the Meningitis Array in Glass Slide Format 

**Figure 2:**
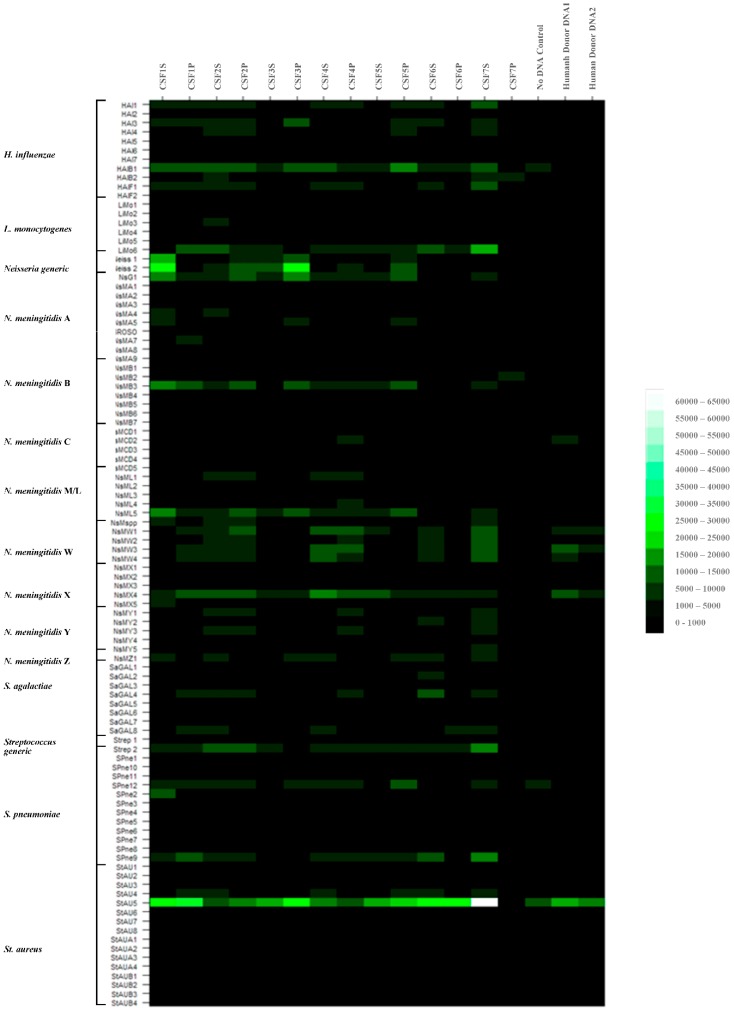
Fluorescence intensity heat-map of probe hybridization using amplified targets from patient cerebral spinal fluid (CSF) nucleic acids, hybridized to the glass slide-printed meningitis microarray using the manual hybridization method and formamide-free hybridization buffer.

Randomly amplified targets from all seven patient CSF samples, supernatant (suffix S) and pellet fractions (suffix P) were amplified, labelled and hybridized to the array as described above. As a control to ascertain the degree of binding of pathogen-specific probes with a nonbacterial pathogen target, randomly amplified targets with no added DNA or human donor whole blood DNA negative controls were also hybridized to the array. Data were normalized to the global median and ranked then according to significance (*p* ≤ 0.2) for all targets. The data for these hybridizations are depicted in heat-map format in Figure 2 and Supplementary Material S6. Certain samples produced data of high fluorescence binding intensity and good confidence e.g., CSF1S, CSF2P, CSF3P, CSF5P. Pathogen-specific signals could be ascribed to these samples with some degree of confidence. Other samples produced data of low fluorescence binding intensity and reasonably good confidence e.g., CSF3S, CSF6P, CSF7S, and CSF7P to which pathogen-specific signals could be tentatively ascribed. The remaining samples produced data of low fluorescence binding intensity and low confidence e.g., CSF1P, CSF2S, CSF4S, CSF4P, CSF5S and CSF6S and no pathogen-specific signals could be assigned. Putative *Neisseria*-specific hybridization profiles were seen with patient CSF samples, CSF1S, CSF2P, CSF3S, CSF3P and CSF5P. However, no definitive serogroup identification could be made in these samples, due to incomplete or mixed serogroup profiles. These showed some of the strongest *Neisseria*-specific profiles. Somewhat weaker microarray binding profiles were also observed in samples CSF6P, CSF7S and CSF7P. No species could be ascribed to the streptococci-containing samples, again due to incomplete strain profiles. It could be seen therefore that a high degree of background nonspecific hybridization was obtained using amplified DNA targets from patient CSF samples.

#### 3.2.2. Further Development of Methods for Pathogen Identification in Patient CSF Samples to the Meningitis Array in Glass Slide Format

**Figure 3:**
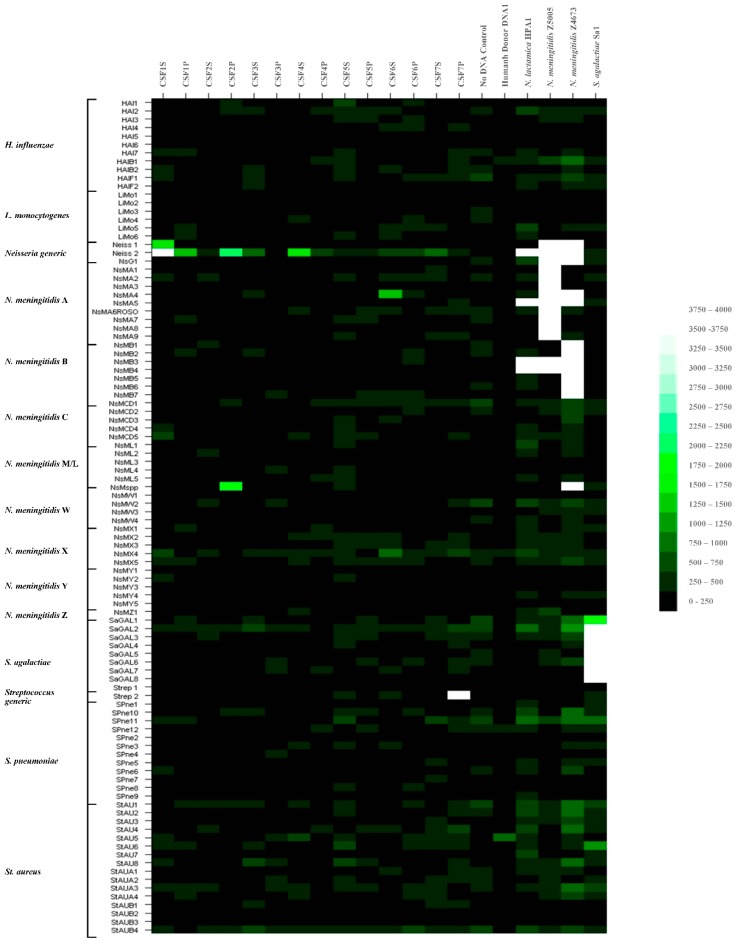
Fluorescence intensity of probe hybridization with amplified targets using patient CSF nucleic acids, depicted in heat-map format. From the glass slide-printed meningitis microarray, hybridizations using the Advalytix hybridization platform and formamide-containing hybridization buffer.

We have shown previously that identification of pathogen-specific identification signatures was achieved using highly purified pathogen nucleic acids and were observed tentatively with some patient CSF DNA samples. This latter result may be due in part to low abundance of the bacterial DNA and high nonspecific background signals from contaminating host DNAs. As the signal-to-noise ratios with our existing hybridization buffers and protocols were not satisfactory, we sought to improve this technical issue through the use of 16% formamide-containing hybridization buffers and an automated hybridization station, the Advalytix Slidebooster™, to improve sample mixing during hybridization. Patient CSF samples were again hybridized to the meningitis glass slide array, (as described in Section 2.6). Samples of no added DNA, human Donor DNA, *N. lactamica* HPA1, *N. meningitidis* strain Z5005 (Serogroup A), *N. meningitidis* strain Z4673 (Serogroup B) and *S. agalactiae* amplified targets were used as negative and positive controls. The results were confirmed for *N. meningitidis* using real-time PCR assay for the *ctrA* gene. The data obtained are shown in Figure 3 and Supplementary Material S7. The combined data for all these hybridizations and real-time PCR results are summarised in Table 1. 

Overall formamide-containing buffers gave lower fluorescence binding intensity signals, across all probes. Addition of 16% formamide reduced overall signal intensities by approximately seven-fold. There was an observed overall improvement in the signal-to-noise ratio and the nonspecific hybridization to previously identified rogue probes e.g., StAU5 was greatly reduced. *N. lactamica*, *N. meningitidis* strains Z5005 and Z4673 and *S. agalactiae* hybridizations gave strong pathogen-specific binding signals to specific oligonucleotide probes, as expected for those sample DNAs. Data of high fluorescence intensity and good confidence were obtained with some samples e.g., CSF1S, CSF1P, CSF2P, CSF4S and CSF7P. Pathogen-specific signals could be ascribed to these samples, with some degree of confidence. Data of low fluorescence intensity and reasonably good confidence were obtained with other samples e.g., CSF3S, CSF4P, CSF6S, CSF6P and CSF7S; pathogen-specific signals could be tentatively ascribed to these samples. The remaining hybridizations produced data of low fluorescence intensity and low confidence e.g., CSF2S, CSF3P, CSF5S and CSF5P; pathogen-specific signals could not be ascribed to these samples with any degree of confidence. 

Thus, putative *Neisseria*-specific hybridization profiles were seen with patient CSF samples, CSF1S, CSF1P, CSF2P, CSF3S, CSF4S and CSF4P, CSF6S and CSF6P and CSF7S. These results corresponded more precisely with the results of the real-time PCR for *N. meningitidis ctrA*, as five out of six real time-PCR (RT-PCR) positive samples were now correctly identified, although some RT-PCR negative samples now appear positive e.g., CSF3S, CSF6P and CSF7S. These discrepancies may be due to the fact that we are working at the limits of detection and there are likely to be sampling errors between different aliquots of the same sample. Tentative serogroup identification could be made in two of the *Neisseria*-containing samples, serogroup X in CSF4P and serogroup C/D in CSF6S and CSF6P, as hybridization to serogroup-specific probes were observed in these samples. A streptococci profile was observed again in CSF7P. No specific species could be assigned for this sample.

### 3.3. Determination of the Infectious Pathogen in Patient CSF Samples Using Multiplex RT-PCR

All clinical samples in the larger secondary panel were assayed using RT-PCR assays for common meningitis-associated pathogens (described in Section 2.9). Results of all RT-PCR-positive samples and the paired associated culture results are given in Table 2. Eighteen CSF samples out of one hundred and forty-six tested (~13%), were found to be positive for bacterial pathogens by RT-PCR. The majority of these were found to contain *S. epidermidis* (10/18, ~54%), *S. epidermidis* and *S. aureus* (1/18, ~5%). The remaining samples were found to contain either *N. meningitidis* (4/18, ~21%), *H. influenzae* (3/18, ~15%) or *S. pneumoniae* (1/18, ~5%). Only 3/18 were positive by bacterial culture, samples 14, 90 and 145 which also exhibited low C_t_ values, indicating a high amount of pathogen nucleic acid and vis á vis the pathogen present.

### 3.4. Redevelopment of the Meningitis Array on the Alere ArrayTubeTM Platform

The meningitis array configured in glass slide format was useful for probe validation; however in this format it has limited use in a routine diagnostic context. We therefore reprinted the best performing probes on the Alere ArrayTubeTM system, using a specific target amplification system to improve the specificity and sensitivity of hybridization. This was evaluated, using amplified, labelled bacterial pathogen DNA targets and a larger cohort of patient CSF samples.

#### 3.4.1. Singleplex and Multiplex Target Amplification Using Purified Pathogen Nucleic Acids and Hybridization to the Meningitis Array in ArrayTube^TM^ Format

**Figure 4:**
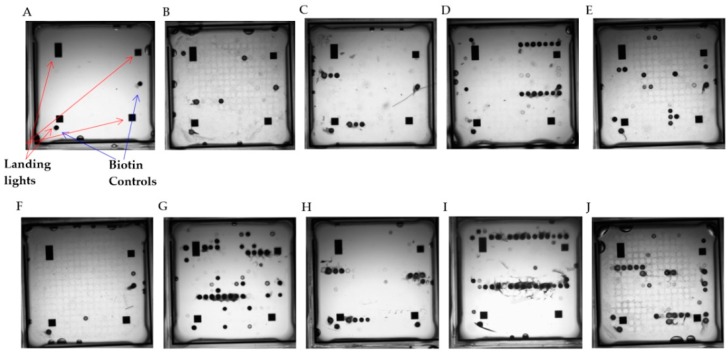
Results of singleplex amplification and microarray hybridization of select, biotin-labelled amplified purified pathogen DNA targets and hybridization to the Alere ArrayTube^TM^ printed meningitis microarray. (**A**) No template control singleplex amplification, (**B**) *Streptococcus pneumoniae* singleplex amplification (Primers SPne1_2, 2.5 ng/mL target DNA), (**C**) *Listeria monocytogenes* singleplex amplification (Primers LiMo4_6, 2.5 ng/mL target DNA), (**D**) *Staphyl aureus* singleplex amplification (Primers StAUB_3, 2.5 ng/mL DNA). (**E**) *N. meningitidis* Serogroup B singleplex amplification (Primers NsMB4, 2.5 ng/mL DNA), (**F**) No template control multiplex amplification, (**G**) *S. pneumoniae* multiplex amplification, (**H**) *L. monocytogenes* multiplex amplification, (**I**) *S. aureus* multiplex amplification, (**J**) *N. meningitidis* serogroup B multiplex amplification.

**Figure 5:**
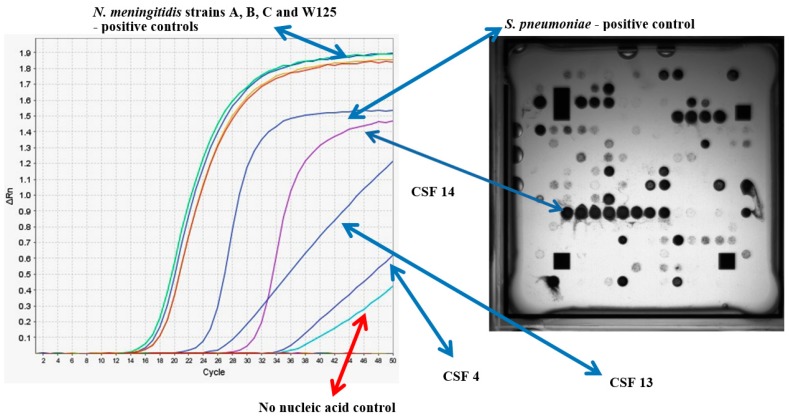
Multiplex RT-PCR (from [82]) for *N. meningitidis* and *S. pneumoniae* with purified pathogen DNA for *N. meningitidis* serogroups A-, B-, C- and W135- and patient CSF samples 4-, 13- and 14- and no template control. An ArrayTube^TM^ multiplex amplification hybridization image for CSF sample 14 is depicted alongside the RT-PCR trace.

Pathogen-specific primer pairs were assessed for amplification of their product in singleplex and analyzed using gel electrophoresis (data not shown). These amplification products were then used in singleplex hybridizations to the ArrayTube^TM^ reconfigured meningitis array (AMA), to confirm the correct probe binding profile for each target. A viable multiplex amplification containing each target primer pair was then constructed (tested at each stage with the requisite bacterial pathogen DNA and using gel electrophoresis). Once a functional multiplex PCR was confirmed, this assay was again used to generate biotin-labelled target amplicons, which were hybridized to AMA tubes, according to the above-described protocol (select single and multiplex hybridization profiles using purified pathogen nucleic acids are given in Figure 4 and Figure 5 and Supplementary Material S9).

The hybridization patterns were as expected for each pathogen for both single and multiplex hybridizations, revealing hybridization of the expected amplified product to the correct probe(s), as expected. However, the multiplex hybridizations again revealed nonspecific binding to rogue probes e.g., HAIB1, MTB3, 4, NsMA5, NsMapp, StAU5, SPne12. A number of these nonspecific hybridization events were seen previously using the glass slide printed arrays. This appears to be a technical issue as a function of multiplex amplification of targets and it is apparent that this is platform independent. Hybridization to these rogue probes was also observed using negative control DNAs, these signals could be filtered out or used to baseline other test hybridization profiles 

During data analyses or the probes removed from the array. Addition of 16% formamide to the glass slide configured array removed this background cross-hybridization; we therefore surmise that these are due to secondary structure binding events of unknown origin.

#### 3.4.2. Multiplex Target Amplification Using Purified Pathogen Nucleic Acids and Hybridization to the Meningitis Array in ArrayTube™ Format Using Patient CSF Samples

**Figure 6:**
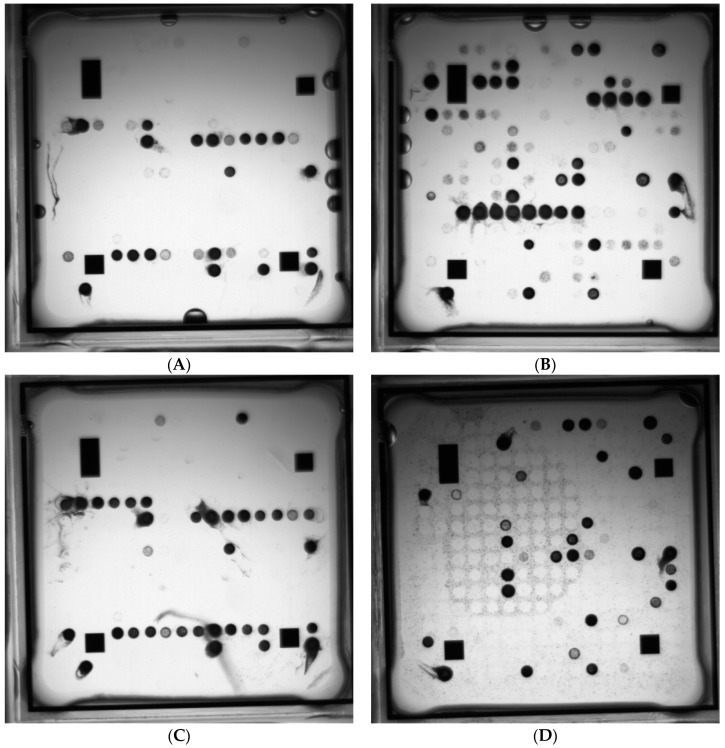
ArrayTube^TM^ multiplex amplification hybridization images for real-time-PCR positive patient CSF samples (results summarized in Table 3). (**A**) Sample 4, (**B**) sample 14, (**C**) sample 72, (**D**) no template control. See Supplementary Material S10.

Microarray hybridization targets were amplified from all bacterial RT-PCR-positive clinical CSFs from Table 2. All clinical sample and negative control hybridizations are given in Supplementary Materials S10 and S11 and are summarised in Table 3 with select hybridizations for samples C4, C14 and C72 in Figure 6. Ten of twenty clinical CSF samples gave results concordant with that of the RT-PCR assays. C4, C13, C72 and C145 were confirmed as *N. meningitidis* positive, three of the strains were identified as serogroup A and one as serogroup B. C4, C29 and C34 were confirmed as *S. epidermidis* and C29 additionally with streptococci pp. C14 was confirmed as *S. pneumoniae* positive, C21 as *H. influenzae* positive and C24 as Group B streptococci positive. Interestingly C54 and C63 showed a hybridization pattern indicative of *S. pneumoniae*, in addition to the expected *S. epidermidis* signal, which may indicate a mixed infection. The *S. pneumoniae* signal was not detected using RT-PCR, but a clear signal could be seen using microarray hybridization. C127 displayed a hybridization pattern for *H. influenzae*. Thus there appeared to be an incomplete correlation between the RT-PCR and microarray hybridization results. The microarray may be detecting pathogen signals in the samples not detected using RT-PCR and vice versa, which may be due to sampling errors.

## 4. Discussion

In this study, we describe development and use of a meningitis diagnostic microarray for specific meningitis-associated pathogen detection, molecular serogrouping of previously identified strains of *N. meningitidis* and for molecular serogrouping of previously unassigned strains. We previously developed a pan-pathogen microarray based on conserved and species-specific bacterial and viral sequences [66,71]. This was shown to be capable of specific pathogen identification using amplified fluorescently labelled targets and to be useful in the discrimination of closely-related *Bacillus* spp. As part of further development to extend the capability of this pan-pathogen array, we have now designed a probe set for detection of five of the most significant ABM pathogens: *N. meningitidis*, *H. influenzae*, *S. agalactiae*, *S. pneumoniae*, *S. aureus* and *L. monocytogenes* and other bacterial species. We also included probes which would discriminate between the *N. meningitidis* serogroups A, B, C, W, X, Y and Z [87]. 

Validation of the ABM probe set in glass slide format using the previously published random amplification method [72] was initially conducted in order to validate the pathogen-specific probes, using a method to limit bias toward known targets during amplification of test and control nucleic acids. From the results presented here we conclude that the meningitis-associated oligonucleotide probe set showed clear, specific hybridization patterns using randomly-amplified pathogen nucleic acids, with manual hybridization methods and formamide-free buffers. Consistent pathogen-specific profiles were observed with both nonmeningococcal and meningococcal pathogens with many of the designed probes, with some evidence of strain differences where more than one strain was tested. A small number of the specific probes did not function well as discussed or showed nonspecific binding to a number of unrelated DNA targets. These nonspecific hybridization signals could be corrected using a human DNA control; however they are not generally useful as diagnostic probes using this hybridization protocol format. However, the limitations of this method are reduced sensitivity i.e., within a mixed pool of nucleic acids (i.e., pathogen and host in clinical CSF samples) the target of interest may be in low concentration. Amplification and subsequent hybridization of nucleic acids amplified equally in this mixed pool reduces the hybridization signal from specific targets of interest to their oligonucleotide probes on the array. This reduces the overall sensitivity of detection. To ascertain whether this could be due in part due to poor mixing during hybridization, we trialled the use of the Advalytix Slidebooster hybridization station, with and without the addition of formamide containing buffers, to investigate whether we could ameliorate these technical issues. 

Pathogen-specific profiles were obtained for *H. influenzae*, *S. agalactiae*, *S. pneumoniae*, *S. aureus*, *L. monocytogenes* and all *N. meningitidis* strains tested. These probe sets were therefore valuable in detection of the relevant organism. Some Neisseria group probes showed binding with many bacterial DNA targets e.g., NsG1, NsMB3 and NsML5 and hybridized to nonpathogenic *Neisseria* spp targets e.g., pilin-related protein probes NsMA4 and NsMB4, suggesting these have homologues in all of the *Neisseria* strains examined. However, most serogroup-specific probes were highly discriminatory and were found to correctly identify all previously serogrouped strains and to ascribe serogroups to previously ungrouped strains. This profile information would be useful in determining the serogroup of meningococcal strains which have no previous serogroup assignation. Other groups have conducted similar studies; Swiderek et al. used a DNA microarray for multilocus sequence typing of *N. meningitidis* [88], Coreless et al. characterized various *N. meningitidis* isolates using resequencing DNA microarrays [61] and Lin used resequencing arrays to identify a range of different respiratory tract pathogens [89]. Previous work has also demonstrated the utility of microarrays to serogroup other bacterial pathogens e.g., *E. coli*, *Shigella* spp. [52,90,91] and streptococci spp. [92,93], to genotype various pathogens associated with infection [48,94,95] and to detect antibiotic resistance genes in *Salmonella* spp. [96] and *S. aureus* [97]. Therefore, these and other microarrays have proven utility for both detection and simultaneous strain profiling of meningitis-associated species and strains and additional functionalities.

Nonpathogenic *Neisseria* spp. including the commensal *N. lactamica*, showed binding profiles to the *Neisseria* generic 16S rRNA probes; these and other probes in this study i.e., NsMA5 and NsMB4 were identified as *Neisseria* generic probes. These nonpathogenic *Neisseria* spp therefore share gene similarities with pathogenic *N. meningitidis* strains. Other groups have shown a general high degree of gene conservation among *Neisseria* isolates, even in genes considered to be associated with pathogenicity [84,88,89,90,98,99,100,101]. Type IV pilin-related proteins have been shown to be present in many *Neisseria* spp including *N. meningitidis* serogroup B [91,92,93], *N. gonorrhoeae* [102] and in the nonpathogenic strain *N. lactamica* [15,16]. Thus a proportion of the *Neisseria* probes were identified as having utility for identification of all *Neisseria* spp, with an additional sub-set for more specific identification of pathogenic *N. meningitidis* strains.

The meningitis-associated probe set was shown to correctly identify pathogen-specific profiles using highly purified pathogen nucleic acid targets. With a view to future implementation of this technology as a routine diagnostic tool, we wished to establish whether this method could be used to identify pathogen signals in CSF samples from patients with suspected meningitis. Randomly-amplified, labelled targets from purified CSF nucleic acids were hybridized to the array using formamide-free manual hybridization methods. Recognisable pathogen signals were detected in eight out of fourteen of the CSF samples tested. Amplified, labelled human nucleic acid cross-hybridized to a significant degree with certain pathogen probes e.g., StAU5 and some of the *N. meningitidis* W and X serogroup-specific probes. Although the background hybridization signals with these samples were high and the signal-to-noise ratio poor, the hybridization patterns were consistent over a number of experiments. Patient CSF targets have been re-amplified and hybridized on a number of occasions with similar results being consistently obtained. These results are most likely due to the presence of background host nucleic acid and a relatively low relative concentration of pathogen nucleic acid in these samples, as evidenced by the high C_t_ values obtained using the real-time PCR assays. These suggest that the concentration of pathogen DNA in these samples is limiting and these diagnostic tests are likely to be operating at the very limits of detection. Improvements in signal resolution were obtained through the use of improved data analysis methods and/or background correction using a human DNA control, as discussed previously. However the presence of human nucleic acids in these samples most probably still occludes the diminishingly small pathogen signal due to its overwhelming abundance in the amplified target. Similar observations have been made using human patient sample materials with other bacterial or viral pan-pathogen arrays [103] As a consequence other groups have developed complex bioinformatics approaches for resolving pathogen microarray signals in a complex mix of host and pathogen nucleic acids [15,44,45,104]. The relatively simple method for data correction to a hybridization control as described was useful for resolving pathogen-specific signals in these CSF clinical samples; however further work would be required to improve the efficacy of this hybridization format method for use in a clinical context.

In addition to improved data analysis methods, we sought to improve hybridization protocols to reduce nonspecific background and improve signal resolution from cross-hybridization events. A number of hybridization refinements were evaluated experimentally in an attempt to improve the signal-to-noise ratio. These included prehybridization and hybridization blocks using tRNA, salmon-sperm, mouse or human DNA and two-colour hybridizations using either human or mouse amplified DNA targets in the second (Cy5) channel. None of these treatments had the desired effect of improving the pathogen-specific signal. In addition, the presence of added foreign DNA was found to block hybridization to probes designed to highly conserved genes from which good fluorescence intensities had previously been obtained e.g., 16S rRNA probes. As an alternative we evaluated the addition of formamide to our existing hybridization buffers and hybridization using an automated hybridization station, the Advalytix Slidebooster^TM^. Various different protocols were tried including varying formamide concentration and incubation time (data not shown), however we found that 16% formamide (*v*/*v*) and six hours of incubation at maximum mix speed MP27 gave optimal results for both pathogen and clinical DNA target hybridizations. 

DNA targets from patient CSF samples CSF1S-CSF7P, controls without DNA, human donor DNA controls, *N. lactamica*, *N. meningitidis* strains Z5005 and Z4673 and *S. agalactiae* were hybridized to the array using the revised protocol. Addition of 16% formamide to the hybridization buffer was found to significantly improve the resolution of the specific signal with all hybridized targets. Automated hybridization methods in our experience appear to reduce nonspecific binding and improve the signal-to-noise ratio. Pathogen-specific signals could be seen in a number of the clinical samples which were detected more readily above background when compared with results obtained using manual hybridizations, despite overall lower signal intensities. Pathogen signals were detected in ten out of fourteen pellet or supernatant CSF samples tested, with clear recognisable signals for Neisseria species in four of ten and Streptococci in one of ten samples tested. Although *Neisseria* spp. profiles were seen, these could not all be ascribed precisely to a strain or serogroup; some indication of serogroup-specific profiles was seen with samples CSF4P (serogroup X), CSF6S and CSF6P (serogroup C/D). However, in the main, the fluorescence intensity of target binding to the serogroup-specific probes with all *N. meningitidis* and CSF amplified targets was lower than to the generic probes e.g., Neiss1 and Neiss2. This may reflect the nature of the genes from which these probes were designed, some genes being present in multicopy e.g., rRNA genes, while others are present only in single copy. Hybridization signals to single copy numbers genes including those for serotyping *N. meningitidis* strains were weaker than those for multicopy genes.

Some strains of *N. meningitidis* do not contain the genes for capsule biosynthesis and would therefore not be readily typed using either gene or protein capsule markers [105]. In addition, other strains may exhibit phase variation of capsule and other outer membrane protein genes [106,107], causing difficulties in classical serotype diagnosis of outbreak strains. These strains would still be detectable and typeable using this microarray. In short, we improved hybridization specificity using formamide-based buffers and the Advalytix Slidebooster hybridization station; however, other technical issues still remain. In reflection this microarray method may not be suitable for use as a front-line diagnostic assay, without further protocol refinements. 

The array was also refocused and transferred to the ArrayTube™ platform (Alere Technologies GmbH, Jena, Germany) to evaluate its use for development of high throughput front-line diagnostics. This system is fully developed and more amenable to routine and high-throughput use than glass slide format. These lower density microarrays offer a viable alternative because they can screen multiple targets simultaneously in a simple high-throughput system [108,109,110,111], are rapid, use inexpensive reagents and would fit seamlessly into clinical laboratories [78,109]. This system was trialled and confirmatory qPCR assays run in parallel for the same ABM targets using published and laboratory developed assays. Transfer of the more focused array to ArrayTube^TM^ format was successful and a proof of concept evaluation using purified pathogen nucleic acids and known positive patient CSF samples showed promise, with clear serotyping profiles obtained for suspected *N. meningitidis* strains. The targeted amplification system appeared to reduce nonspecific background hybridization signals from human contaminating nucleic acids, thus overcoming some of the problems encountered using the random amplification method. Some similar technical issues arising in the previous hybridization protocol formats were observed here also, in particular nonspecific hybridization to certain probes. These occurred consistently in sample and paired negative controls. For future use removal of these rogue probes would be relatively straightforward, either during data processing or physically from the array. The parameters for true positives explored by Batchelor et al. [78] and in [112] were useful as a reliable method for setting cut-off values and enabled data processing and identification of significant pathogen probe profiles. The colorimetric detection protocol is also faster and more efficient [113] and would allow for higher-throughput sample processing and detection [109], a critical feature for use within a clinical setting. Although some sensitivity issues remained at low pathogen concentrations (as evidenced by high real-time-PCR C_t_ values) and modification of the protocol to include a targeted amplification system, the ABM microarray format shows some promise as a diagnostic tool within a clinical setting. Use of this pan-bacterial meningitis array could facilitate simultaneous detection of diverse targets, negating the use of multiple separate assays, leading to improvements in time to result in comparison to other molecular diagnostic tests. However, there are some limitations and the outputs do not correspond precisely with those obtained by RT-PCR. This format does show some promise for parallel and rapid detection of meningitis-causing pathogens, however further development work would be required.

In summary, we aimed to develop a diagnostic test hybridization system based on generic amplification of targets which would not require any prior knowledge of the organism and which would not slant bias toward known pathogens. This was with a view to establishing a method that could be used with either multiplex pathogen-specific pre-designed array sets or generic arrays such as the noncognate arrays developed by Schrenzel and colleagues [42]. Many groups have designed microarrays for detection of pathogens in clinical and environmental samples. However in the main, these have utilized methods that amplify and hybridize specific PCR products complementary to the pathogen-specific probes represented on the array. The FluChip microarray for detection of influenza virus is one example of this microarray type and has already been validated for use in routine disease diagnosis [114]. Other similar technologies include resequencing arrays which have been shown to be useful for identification of pathogens [83,115], recombination microarrays to detect novel viruses [116] and those that may be adapted for automated high-throughput pathogen detection [117]. Although we chose deliberately to use the random-amplification method to reduce bias in generating targets from material which may contain unknown pathogens, the limitation of the method is that it amplifies all nucleic acids in the sample as well as those of any pathogen present. Overall, the array using randomly-amplified targets showed good utility in detecting pathogen-specific signals using purified pathogen nucleic acids, but was less efficient using clinical CSF sample material due to the technical limitations outlined above. 

Transfer to the ArrayTube^TM^ platform format and the use of specific amplification methods showed the best potential with respect to generation of pathogen specific signals with the requisite specificity and sensitivity of hybridization, as required for a routine diagnostic tool. The ability of the assay to be rapid, efficient, specific and sensitive is essential for establishment of a robust assay for routine use and this array may not be useful in that context without considerable further protocol refinement. However, this would require more extensive optimization and at this present time its use is most likely limited to species and strain typing from culture, within a laboratory setting. It could be useful in improving detection of diverse targets in meningitis in a laboratory setting using purified pathogen nucleic acids, similar to other Alere ArrayTube^TM^ systems for detection and typing of pathogens [118]. This test may facilitate more rapid species and strain designation, thus improving time to diagnosis and improving clinical outcome. Similar types of assays have been developed using Loop-Mediated isothermal Amplification methods [119], however these are run as independent, single-tube reactions at present and currently lack multiplexing capacity.

## 5. Conclusions

Validation of the meningitis microarray in glass slide format, using randomly amplified DNA targets, with and without the use of formamide-containing buffers was successful. However, there were some technical issues with nonfunctioning and cross-hybridizing probes to bacterial and human DNA control targets. This and the long and complex hybridization protocol not readily amenable to high-throughput, limits the use and ongoing development of the array in this format. Repurposing the array onto the Alere ArrayTube^TM^ format and using a targeted amplification system was fruitful and clear pathogen-specific signals could be seen with pathogen DNA and clinical CSF sample hybridizations. This would lend itself useful as a routine diagnostic tool in a laboratory setting using purified pathogen nucleic acids, with the additional capability for *N. meningitidis* serogroup identification.

## Figures and Tables

**Table 1:** Details of processed patient cerebrospinal fluid samples and positivity for *Neisseria meningitidis* using real-time polymerase chain reaction (RT-PCR) and microarray hybridization.

Patient	Sample Designation		Sample Fraction	Culture	RT-PCR C_t_ Values for *Neisseria meningitides* (*ctrA*) *	Microarray Signal for *Neisseria* spp.
1	CSF1S	Cerebrospinal Fluid	supernatant	-	36	+
1	CSF1P	Cerebrospinal Fluid	pellet	-	37	+
2	CSF2S	Cerebrospinal Fluid	supernatant	-	35	-
2	CSF2P	Cerebrospinal Fluid	pellet	-	31	+
3	CSF3S	Cerebrospinal Fluid	supernatant	-	ND	-
3	CSF3P	Cerebrospinal Fluid	pellet	-	ND	-
4	CSF4S	Cerebrospinal Fluid	supernatant	-	ND	+
4	CSF4P	Cerebrospinal Fluid	pellet	-	38	-
5	CSF5S	Cerebrospinal Fluid	supernatant	-	ND	-
5	CSF5P	Cerebrospinal Fluid	pellet	-	ND	-
6	CSF6S	Cerebrospinal Fluid	supernatant	-	36	-
6	CSF6P	Cerebrospinal Fluid	pellet	-	ND	-
7	CSF7S	Cerebrospinal Fluid	supernatant	-	ND	-
7	CSF7P	Cerebrospinal Fluid	pellet	-	ND	-

* *CtrA* RT-PCR conducted at PHES. ND: not detected.

**Table 2:** Details of RT-PCR bacterial pathogen positive patient cerebrospinal fluid samples.

Sample Number	M/F	Date of Sample	Syndromic Meningitis PCR (CSF Unless Stated)	Bacterial Culture	White Blood Cells 10^9^/L	CRP mg/L	Other/Clinical/Travel History
4	F	25 June 2011	Ent, HSV1/2, VZV-ve	no growth	4	<1	
11	M	4 July 2011	*N. meningitidis*-ve	no growth	11.7	16	
13	F	1 July 2011	Ent, HSV1/2, VZV-ve	N/A	N/A	N/A	
14	F	5 July 2011	Ent, HSV1/2, VZV-ve	*S. pneumoniae*	9.5	185	clinical details: “pyrexia-strep meningitis”; β haemolytic *Streptococcus* not isolated
21	F	22 July 2011	Ent, HSV1/2, VZV-ve	N/A	N/A	N/A	
24							
29	F	21 July 2011	*Borrelia burgdorferi*-ve	N/A	N/A	N/A	
34	M	28 July 2011	Ent, HSV1/2, VZV-ve	N/A	N/A	N/A	
51	F	30 March 2011	Ent, HSV1/2, VZV, *N. meningitidis*-ve	no growth	7.8	13	
54	F	4 April 2011	Ent, HSV1/2, VZV, *N. meningitidis*-ve	no growth	18.6	<1	headache, photophobia, recent trip to Gambia
61 *	M	13 April 2011	AdV, RotaVirus, Ent, HSV1/2, *N. meningitidis*-ve	no growth	10	6	
63 *	M	13 April 2011	AdV, RotaVirus, Ent, HSV1/2, *N. meningitidis*-ve	no growth	64	6	
70	F	20 April 2011	Ent, HSV1/2, VZV-ve	no growth	6	10	
72	U	14 April 2011	*B. burgdorferi*-ve	N/A	N/A	N/A	
76	M	27 April 2011	Ent, HSV1/2, VZV-ve	no growth	13.3	2	
90	F	12 May 2011	Ent, HSV1/2, VZV-ve	mixed perineal flora	14.3	55	Pyrexia of unknown origin
95	M	16 May 2011	Ent, HSV1/2, VZV-ve	no growth	6.8		
127	M	23 June 2011	Ent, HSV1/2, VZV, *N. meningitidis*-ve	no growth	<1		
145	F	14 July 2011	Parvo, CMV, EBV-ve (CSF). *N. meningitidis*-ve (blood) Varicella IgG detected (blood)	*N. meningitidis* in blood bottle		60	Ref lab report: Type B, subtype P1.7/P1.1/NT; porA seq: 7-1/1/35-1
147	F	4 June 2011	Ent, HSV1/2, VZV, *N. meningitidis*-ve	no growth	<1	7	

CRP: C-reactive protein, N/A: not available, * repeat sample from same patient.

**Table 3:** RT-PCR Values of Bacterial Spp.-Positive Clinical Samples and Corresponding Array Result. See Supplementary Material S11.

Sample Number	Bacterial RT-PCR Result	C_t_ Value	Microarray Hybridization Result Multiplex	Probable Strain Designation
4	*Neisseria meningitidis*	43.72	*N. meningitidis*	*N. meningitidis* A
11	*Staphylococcus epidermidis*	43.28	*N. meningitidis*/*Staphylococcus* spp.	unknown
13	*Neisseria meningitidis*	39.88	*N. meningitidis*	*N. meningitidis* A
14	*Streptococcus pneumoniae*	31.85	*S. pneumoniae* *	
21	*Haemophilus influenzae*	37.34	ND	
24	Group B Streptococci	36.39	ND	
29	*Staphylococcus epidermidis*	36.48	ND	
34	*Staphylococcus epidermidis*	38.8	*S. pneumoniae* *	
51	*Staphylococcus epidermidis*	39.37	*L. monocytogenes* *	
54	*Staphylococcus epidermidis*	38.79	*S. pneumoniae*	
61	*Staphylococcus epidermidis*	37.59	*N. meningitidis*	*N. meningitidis* A *
63	*Staphylococcus epidermidis*	40.66	*S. pneumoniae*/*Staphylococcus* spp. *	
70	*Haemophilus influenzae*	40.36	ND	
72	*Neisseria meningitidis*	36.00	*N. meningitidis*	*N. meningitidis* A
76	*Staphylococcus epidermidis* & *Staphylococcus aureus*	37.87, 45.13	ND	
90	*Staphylococcus epidermidis*	39.02	ND	
95	*Haemophilus influenzae*	36.02	*S. pneumonia* *	
127	*Staphylococcus epidermidis*	40.19	Neisseria spp.	
145	*Neisseria meningitidis*	19.38	*N. meningitidis*/*M. tuberculosis*	*N. meningitidis* B
147	*Staphylococcus epidermidis*	40.36	ND	

ND: Not detected or indeterminate. * Denotes a weak signal.

## Supplementary Materials

The following are available online at http://www.mdpi.com/2571-5135/7/4/32/s1; Supplementary Material S1; Table S1: ‘Bacterial Species and Strains used in Study’. Supplementary Material S2; Tables S2 and S3: ‘Microarray Oligonucleotide Probes used in Study’, ‘Table S3: ‘Additional Microarray Oligonucleotide Probes used in Study’. Supplementary Material S3; Table S4: ‘List of PCR Primers for Target Amplification used in Study’. Supplementary Material S4; Table S5: ‘List of PCR Primers and probes for Real-Time PCR used in Study’. Supplementary Material S5; Table S6: ‘All normalized Cy3 Fluorescence intensity raw data values’, Table S7: ‘All normalized Cy3 Fluorescence intensity raw data calculated *p* values’, Table S8: ‘All Top Ranked Probe Hits (*p* ≤ 0.2)’, Figure S1: *H. influenzae* HPA, Figure S2: *H. influenzae* Hi2, Figure S3: *L. monocytogenes*, Figure S4: *S. pneumoniae* NCTC07465, Figure S5: *Str agalactiae* Sa1, Figure S6: *S. aureus* EMRSA-16, Figure S7: *S. aureus* NCTC29213, Figure S8: *N. meningitidis* Z5005, Figure S9: *N. meningitidis* Z4673, Figure S10: *N. meningitidis* Z8948, Figure S11: *N. meningitidis* Z6904, Figure S12: *N. meningitidis* NCTC9714, Figure S13: *N. meningitidis* NCTC11423, Figure S14: *N. meningitidis* NCTC11424, Figure S15: *N. meningitidis* NCTC10790, Figure S16: *N. meningitidis* NCTC10791, Figure S17: *N. meningitidis* NCTC10792, Figure S18: *N. meningitidis* NCTC10793, Figure S19: *N. meningitidis* NCTC13152, Figure S20: *N. meningitidis* NCTC13198, Figure S21: *N. meningitidis* NCTC13214, Figure S22: *N. meningitidis* NCTC13218, Figure S23: *N. meningitidis* NCTC13223, Figure S24: *N. meningitidis* NCTC13225, Figure S25: *N. meningitidis* NCTC13248, Figure S26: *N. meningitidis* NCTC13273, Figure S27: *N. meningitidis* NCTC13275, Figure S28: *N. lactamica* HPA1, Figure S29: *N. lactamica* Y92-1009, Figure S30: *N. lactamica* 020-06, Figure S31: *N. flavescens*. Supplementary Material S6; Table S9: ‘All normalized Cy3 Fluorescence intensity values’, Table S10: ‘All normalized Cy3 Fluorescence intensity calculated *p* values’, Table S11: ‘All Top Ranked Probe Hits (*p* ≤ 0.2)’, Figure S32: CSF1S, Figure S33: CSF1P, Figure S34: CSF2S, Figure S35: CSF2P, Figure S36: CSF3S, Figure S37: CSF3P, Figure S38: CSF4S, Figure S39: CSF4P, Figure S40: CSF5S, Figure S41: CSF5P, Figure S42: CSF6S, Figure S43: CSF6P, Figure S44: CSF7S, Figure S45: CSF7P, Figure S46: No DNA Control, Figure S47: Human Donor DNA 1, Figure S48: Human Donor DNA 1. Supplementary Material S7; Table S12: ‘All normalized Cy3 Fluorescence intensity values’, Table S13: ‘All normalized Cy3 Fluorescence intensity calculated *p* values’, Table S14: ‘All Top Ranked Probe Hits (*p* ≤ 0.2)’, Figure S49: CSF1S, Figure S50. CSF1P, Figure S51: CSF2S, Figure S52: CSF2P, Figure S53: CSF3S, Figure S54: CSF3P, Figure S55: CSF4S, Figure S56: CSF4P, Figure S57: CSF5S, Figure S58: CSF5P, Figure S59: CSF6S, Figure S60: CSF6P, Figure S61: CSF7S, Figure S62: CSF7P, Figure S63: No DNA Control, Figure S64: Human Donor DNA 1, Figure S65: *N. lactamica* HPA1, Figure S66: *N. meningitidis* Z5005, Figure S67: *N. meningitidis* Z4673, Figure S68: *S. agalactiae*. Supplementary Material S8; Table S14: Patient cerebrospinal fluid samples CSF 1 to 7, nomenclature of processed samples and associated nucleic acid concentrations. Supplementary Material S9; Tables S15–S24: ‘All AlereTube^TM^ hybridization profiles; ranked on confidence and then signal intensity (cells with values above the threshold cutoff > 0.4 highlighted in bold text)’, Table S15: Singleplex PCR no template control, Table S16: *S. pneumoniae* singleplex primer pair for microarray probes SPne1 and SPne2 (ply), Table S17: *L. monocytogenes* singleplex primer pair for microarray probes LiMo4 to LiMo6 (hly), Table S18: S. aureus singleplex primer pair for microarray probes StAUB1 to StAUB4 (spa), Table S19: *N. meningitidis* serogroup B singleplex primer pair for microarray probe NsMB4 (ubiA), Table S20: Multiplex PCR no template control, Table S21: *S. pneumoniae* multiplex (all S. pneumoniae probes), Table S22: *L. monocytogenes* multiplex (all *L. monocytogenes* probes), Table S23: *S. aureus* multiplex (all *S. aureus* probes), Table S24: *N. meningitidis* serotype B multiplex (all *N. meningitidis* serotype B probes). Supplementary Material S10; All meningitis Alere ArrayTube multiplex target amplification hybridization images for RT-PCR pathogen-positive patient cerebrospinal fluid samples. Supplementary Material S11; Tables S25–S45: ‘All ranked AlereTube^TM^ hybridization profiles’, Table S25: No template control, Table S26: CSF sample 4, Table S27: CSF sample 11, Table S28: CSF sample 13, Table S29: CSF sample 14, Table S30: CSF sample 21, Table S31: CSF sample 24, Table S32: CSF sample 29, Table S33: CSF sample 34, Table S34: CSF sample 51, Table S35: CSF sample 54, Table S36: CSF sample 61, Table S37: CSF sample 63, Table S38: CSF sample 70, Table S39: CSF sample 72, Table S40: CSF sample 76, Table S41: CSF sample 90, Table S42: CSF sample 95, Table S43: CSF sample 127, Table S44: CSF sample 145, Table S45: CSF sample 147
