# Supplementary material for: Development and Assessment of a Diagnostic DNA Oligonucleotide Microarray for Detection and Typing of Meningitis-Associated Bacterial Species
Source: High Throughput. 2018 Oct 16;7(4):32. doi: 10.3390/ht7040032 (PMC6306750; doi:10.3390/ht7040032)
Supplement: Supplementary file 1 [file high-throughput-07-00032-s001.zip › Supplementary Material S1.pdf]

Supplementary Material S1 - Table S1

**Bacterial Species**	**Serogroup/ Strain**	**Source**
Haemophilus influenzae	HPA	Dr Nigel Silman, HPA, Porton Down, Salisbury
Haemophilus influenzae	Clinical isolate Hi2	Dr. S. Green & Dr. P. Marsh, HPA, Southampton
Listeria monocytogenes	Clinical isolate Lm1	Dr. S. Green & Dr. P. Marsh, HPA, Southampton
Neisseria flavescens	Not typed	Prof. A. Gorringe
Neisseria lactamica	020-2006	Prof. A. Gorringe
Neisseria lactamica	Y92-1009	Prof. A. Gorringe
Neisseria lactamica	HPA1	Prof. A. Gorringe
Neisseria meningitidis	Z5005 (B40) Serogroup A	Prof. A. Gorringe
Neisseria. meningitidis	Z4673 (B2198) Serogroup B	Prof. A. Gorringe
Neisseria meningitidis	Z8948 (FAM18) Serogroup C	Prof. A. Gorringe
Neisseria meningitidis	Z6904 (ROY) Serogroup W135	Prof. A. Gorringe
Neisseria meningitidis	D/ NCTC9714	NCTC, Colindale London
Neisseria meningitidis	X/ NCTC10790	NCTC, Colindale London
Neisseria meningitidis	Y/ NCTC10791	NCTC, Colindale London
Neisseria meningitidis	Z/ NCTC10792	NCTC, Colindale London
Neisseria meningitidis	Z-prime/ NCTC10793	NCTC, Colindale London
Neisseria meningitidis	73-M6/ NCTC11423	NCTC, Colindale London
Neisseria meningitidis	74-M24/ NCTC11424	NCTC, Colindale London
Neisseria meningitidis	NCTC13152	NCTC, Colindale London
Neisseria meningitidis	NCTC13198	NCTC, Colindale London
Neisseria meningitidis	NCTC13214	NCTC, Colindale London
Neisseria meningitidis	NCTC13218	NCTC, Colindale London
Neisseria meningitidis	NCTC13223	NCTC, Colindale London
Neisseria meningitidis	NCTC13248	NCTC, Colindale London
Neisseria meningitidis	NCTC13273	NCTC, Colindale London
Neisseria meningitidis	NCTC13275	NCTC, Colindale London
Neisseria meningitidis	NCTC13225	NCTC, Colindale London
Staphylococcus aureus MRSA	EMRSA-16	Dr. S. Green & Dr. P. Marsh, HPA, Southampton
Staphylococcus aureus MSSA	ATCC29213	Dr. S. Green & Dr. P. Marsh, HPA, Southampton
Streptococcus agalactiae	Clinical isolate Sa1	Dr. S. Green & Dr. P. Marsh, HPA, Southampton
Streptococcus pneumoniae	NCTC07465	Dr. S. Green & Dr. P. Marsh, HPA, Southampton
