# Supplementary material for: Development and Assessment of a Diagnostic DNA Oligonucleotide Microarray for Detection and Typing of Meningitis-Associated Bacterial Species
Source: High Throughput. 2018 Oct 16;7(4):32. doi: 10.3390/ht7040032 (PMC6306750; doi:10.3390/ht7040032)
Supplement: Supplementary file 1 [file high-throughput-07-00032-s001.zip › Supplementary Material S2.pdf]

Supplementary Material S2, Table S2: Microarray Oligonucleotide Probes used in Study

Probe	Organism	Gene	Sequence
HAI1	*Haemophilus influenzae*	***nucA***	TAAATGGTCAGCAAACCAAAGTGGATATTGGTGGTTTTTCTGCTGTCAATGCAAAACTTAACAAATTGCG
HAI2	*Haemophilus influenzae*	***nucA***	GCACAAATTATGGCAAATGCGCTAAAACAGCAAGGAATTAATAAAATTATCCTACTTTCACACGCAGGTA
HAI3	*Haemophilus influenzae*	***nucA***	TTTATTGCAGAAACAATGTATAACGAACTCAAAACAGTGGATTTAACTATTCAAAATGCTGGCGGTGTAC
HAI4	*Haemophilus influenzae*	***nucA***	GAAACACCAAATGCGGAAGGTAAGCGTTTAGTGAGTGTTGAAGTCTTGAATAAACAAACCCAACAATGGG
HAI5	*Haemophilus influenzae*	***cpdB***	TTGATTGCAACGAATAACTATCGTGCTTACGGCAATAAATTCCCAGGTACTGGTGATAAACATATTGTTT
HAI6	*Haemophilus influenzae*	***cpdB***	ATCCAAATGCGGATAAAAACTGGCGTTTTGTGCCTATCACAGGTAACGATAAATTAGATGTCCGTTTTGA
HAI7	*Haemophilus influenzae*	***cpdB***	ATTAACCCAGAATCGCATCGTGTAGTGAATCTCACTTATCAAGGCAAACCAGTTGATCCAAAAGCAGAAT
HAIB1	*Haemophilus influenzae*	***bexA***	TCGGGATTTTAGGGCGAAATGGTGCTGGTAAATCCACGCTCATTCGTTTGATGAGTGGTGTTGAGCCTCC
HAIB2	*Haemophilus influenzae*	***bexA***	AATTGATGAGGTGATTGCAGTAGGGGATTCGCGCTTTGCAGAAAAATGTAAGTATGAGTTATTTGAGAAA
HAIF1	*Haemophilus influenzae*	***bexA***	TTGGGATCCTAGGACGAAATGGAGCAGGCAAATCAACACTTATTCGGTTGATCAGTGGAGTAGAACCCCC
HAIF2	*Haemophilus influenzae*	***bexA***	GATCGATGAAGTAATAGCGGTGGGAGATTCTCGGTTTGCAGAGAAATGCAAATATGAATTATTTGAAAAG
LiMo1	*Listeria monocytogenes*	***dltA***	ATATGTCTCCCCTAATGTTAGTTGCATTCTTAGGCTCTATCAAATCAGGTCGAGCTTATGTACCAGTAGA
LiMo2	*Listeria monocytogenes*	***dltA***	AAGCTTACCACTTGGCGTTATTAAACCAGATATGCGTTTACACATCGTTGACCAAGAAACTGGTGAGGTT
LiMo3	*Listeria monocytogenes*	***dltA***	ATATGCGTTTACACATCGTTGACCAAGAAACTGGTGAGGTTCTTCCAGAAGGCGAAAAAGGAGAAATTGT
LiMo4	*Listeria monocytogenes*	***hly***	TGTAAATAATAGCTTGAATGTAAACTTCGGCGCAATCAGTGAAGGGAAAATGCAAGAAGAAGTCATTAGT
LiMo5	*Listeria monocytogenes*	***hly***	AGTCCTAAGACGCCAATCGAAAAGAAACACGCGGATGAAATCGATAAGTATATACAAGGATTGGATTACA
LiMo6	*Listeria monocytogenes*	***hly***	AATACATTAGTGGAAAGATGGAATGAAAAATATGCTCAAGCTTATCCAAATGTAAGTGCAAAAATTGATT
Neiss 1	*Neisseria* spp.	***16S rRNA***	TTGCGTTATTCGAGCGGCCGATATCTGATTAGCTAGTTGGTGGGGTAAA
Neiss 2	*Neisseria* spp.	***16S rRNA***	TGCTAATACCCGCGGCTGATGACGGTACCTGAAGAATAAGCACCGGCTAA
NsG1	*Neisseria gonorrhoeae*	**type IV PRP**	TATAGTGTTTCGGTGCAGTTTGTCGATGCGGAAAAACCAAGGGCATACAGGTTGGTCGGTGTTCCGAACG
NsMA1	*Neisseria meningitidis* A	***ctrA***	AAGTTAAAGTTTTGTATTGTAATTTCTTTTTTAATTTTGGGAAGTGCATGTAGTGCTATTCCCTCCTCTG
NsMA2	*Neisseria meningitidis* A	***sacB***	CAAAGAATTTTAAAAATTCATCACATATCCATAAAACTAATATAAGTAAAGCTCAATCAAATATTTCTTC
NsMA3	*Neisseria meningitidis* A	***sacB***	GCATGGCTAGATTTAAATAACCCTAAAATTCAATGGGTATATCACGAAGAAATTATGCCACAAAGTGCCC
NsMA4	*Neisseria meningitidis* A	**type IV PRP**	TATAATGTTTCGGTGCATTTTGTCAATGAGGAAAAACCAAGGGCATACAGCTTGGTCGGCGTTCCAAAGA
NsMA5	*Neisseria meningitidis* A	***ubiA***	AATGCGCAGTGCCGGTTGCGTCATCAACGATTTTGCCGACCGCGATTTTGACGGTGCTGTCGAGCGCACC
NsMA6 ROSO	*Neisseria meningitidis* A	***sacB***	GACAGATTTATTTAGCATTGCTCATGTTGACATGAAACTCAGCACAGATAGAACTTTAAGTTCATCTATA
NsMA7	*Neisseria meningitidis* A	***sacB***	CAGTACATCAAGATTCCTTAGTAGAGATGAATTAAAATTCGCATTACGCTCTTGGGAAATGAGTGGATCC
NsMA8	*Neisseria meningitidis* A	***sacB***	GATTCCTTAGTAGAGATGAATTAAAATTCGCATTACGCTCTTGGGAAATGAGTGGATCCTTCATTCGAAA
NsMA9	*Neisseria meningitidis* A	***sacB***	ACGCAATAGGTGTATATATTCTTCCTTCTAATCTTACTCTTAAGCCTGCATTATGTATTCTAGAATCACA

NsMB1	*Neisseria meningitidis* B	***SiaD***	TTATCTTACCCCCCCACGTAACAATTTATTTGTCATATCTAATTTAGGTCAGCTTAACCAAGTCCAAAGC
NsMB2	*Neisseria meningitidis* B	***SiaD***	GAGAGTTAATTATTAACTTAATTCAAAAATATTCAATGGTGGAAAACACTGAAATGATCCAAGAACACTT
NsMB3	*Neisseria meningitidis* B	**type IV PRP**	TATAGTGTTTCGGTAAAGTTTGTCGATAAGGAAAAATCAAGGGCATACAGGTTGGTCGGCGTTCCGAAGG
NsMB4	*Neisseria meningitidis* B	***ubiA***	AATGCGCAGTGCCGGCTGCGTCATCAACGACTTTGCCGACCGCGATTTTGACGGTGCTGTCGAGCGTACA
NsMB5	*Neisseria meningitidis* B	***SiaD***	AACATCTCCATTTTATCTTACCCCCCCACGTAACAATTTATTTGTCATATCTAATTTAGGTCAGCTTAAC
NsMB6	*Neisseria meningitidis* B	***SiaD***	CTAACAAGAATCTATTTGAATCTATTTATCTATTTGAGCTTCCTAGAAGCCCTAATAATATAACTCCTAA
NsMB7	*Neisseria meningitidis* B	***SiaD***	CAATCAGCTAACAAGAATCTATTTGAATCTATTTATCTATTTGAGCTTCCTAGAAGCCCTAATAATATAA
NsMCD1	*Neisseria meningitidis* C/D	***SiaD/SynC***	TTATTATTTCTACATTTGCCCAACTTAATCAAGCTCATAGTCTGACTAAGATGCAAAAATTAAAAAATAAT
NsMCD2	*Neisseria meningitidis* C/D	***SiaD/SynC***	GGCAAATCGTGATTGATTCGATACACCATACAGCCCAACAAGAGAACACCGCTTTAATTGAAGAGCATTT
NsMCD3	*Neisseria meningitidis* C/D	***SiaD/SynC***	AAGATTCCCAGTGGTTTGCAACACCTTTATTTAGCAGCTTCGCACCCAAAAGCAATCTTTTTATTATTTC
NsMCD4	*Neisseria meningitidis* C/D	***SiaD/SynC***	CTCAAAACTATCCAACCTGCACACCTTTATGTTATGTCTTTTGCTGGGCATTATTCCTCTCTGCTCAGCT
NsMCD5	*Neisseria meningitidis* C/D	***SiaD/SynC***	GGGATTAGCACAAGCCAATCTATTGCTAAAATTCAAGACAAATACCGCATATCTCAAAATGACTATATTT
NsML1	*Neisseria meningitidis* L	***lcbA***	GAAATCGAAGAACCGGCCATCATTGCCAATAGCTTGCATTTAGCAGCTGTAGAAAGTGCCATCCATCTTA
NsML2	*Neisseria meningitidis* L	***lcbA***	GATGTTTTTGTAGCACGCCCATTGCATAGGGAACATTTTTTCCACGCAAACGGAATAGCCTCCCTTTTCA
NsML3	*Neisseria meningitidis* L	***lcbA***	GGTGACGCTGTTGCCATTGAGTATCACTATTATCTACCCACGTGAAAACGACATCAATCTTAAATGGGCT
NsML4	*Neisseria meningitidis* L	***lcbA***	TCACGTGGGTAGATAATAGTGATACTCAATGGCAACAGCGTCACCAACAATACTGCCATGCAGCCTCGCC
NsML5	*Neisseria meningitidis* L	***lcbA***	ACGGGTTTCGTCGTTGCTGTACAGATTGTTAGGCGAGGCTGCATGGCAGTATTGTTGGTGACGCTGTTGC
NsMspp	*Neisseria meningitidis*	***CtrA***	TATATTCGTCACGCAGTATTATTATTGTGTGGAAGTTTAATTGTAGGATGCTCTGCGATTCCTTCATCAG
NsMW1	*Neisseria meningitidis* W	***SynG***	TCAGAAAGTGAGGGATTTCCATATATATTTATGGAAGGCATGGTGTATGATATTCCAATCGTTGTATATG
NsMW2	*Neisseria meningitidis* W	***SynG***	TGGAGCGAATGATTACAGTAACTATAATGAAAATGGTTGTGTTTTTAAAACTGGTGATATTTCTGGAATG
NsMW3	*Neisseria meningitidis* W	***SynG***	AGAAAGTGAGGGATTTCCATATATATTTATGGAAGGCATGGTGTATGATATTCCAATCGTTGTATATGAT
NsMW4	*Neisseria meningitidis* W	***SynG***	GACATCAGAAAGTGAGGGATTTCCATATATATTTATGGAAGGCATGGTGTATGATATTCCAATCGTTGTA
NsMX1	*Neisseria meningitidis* X	***XcbB***	ACAAATAGTCGCTTAAACATTATTAAATTAGCCCGAAGAGATGCCCAAGCTTACGATCAATATATTGAAA
NsMX2	*Neisseria meningitidis* X	***XcbB***	ATTATTGACTCATCGGTTTATAATCAAAAAGATGTCCATTTCGTCAAACATTTTAAAGAAGCCAACATGA
NsMX3	*Neisseria meningitidis* X	***XcbA/B***	ACTTAGCGGATGTCACCCTCACATCACAGCCTAGTGGACAATAATAATCTAAGTTACGGAGTAATCGTAT
NsMX4	*Neisseria meningitidis* X	***XcbB***	ACTGAAGGCTTACAGCGATTTGCGCCGCCATAAAGAAATGTTATACACACTGACACCACCTTCACCAAAA
NsMX5	*Neisseria meningitidis* X	***XcbB***	GATTACGAAGACCAGATTCAGAGCCTGATTAAAGAAACCTTATACAAATGCGACATCCAGCCTGAAAACG
NsMY1	*Neisseria meningitidis* Y	***SynF***	TCTCAAAGCGAAGGCTTTGGTTATATATTTCTAGAGGGTATGGTGTACGATATCCCTATCCTTGCCTATA
NsMY2	*Neisseria meningitidis* Y	***SynF***	TGGAGCGAATGATTTTAGCAATTATAATGAAAACGCTTCAGTTTTTAAAACTGGTGATATTTCTGGAATG
NsMY3	*Neisseria meningitidis* Y	***SynF***	GACATCTCAAAGCGAAGGCTTTGGTTATATATTTCTAGAGGGTATGGTGTACGATATCCCTATCCTTGCC
NsMY4	*Neisseria meningitidis* Y	***SynF***	CAAAGCGAAGGCTTTGGTTATATATTTCTAGAGGGTATGGTGTACGATATCCCTATCCTTGCCTATAATT
NsMY5	*Neisseria meningitidis* Y	***SynF***	GAAGGCTTTGGTTATATATTTCTAGAGGGTATGGTGTACGATATCCCTATCCTTGCCTATAATTTTAAAT

NsMZ1	*Neisseria meningitidis* Z	***CtrA***	ATATCTTCTTTTATGATTTATGCGGTGCTGTTCGCTATGGTTACGGCATGTAGTGCTATTCCCTCCTCCG
SaGAL1	*Streptococcus agalactiae*	***pscB***	AGATGTTAGCACAACAAGAGGCTGACAAAGCGGCCTTAGAAGCTAAACAAATTGAAAATCAAAATGCTAT
SaGAL2	*Streptococcus agalactiae*	***pscB***	ACAAGCAAGCTATCGAAAATAATAAAGCAGCCTTAGCAACACAAAGAGCACAATTAGAAGCAGCTCAATT
SaGAL3	*Streptococcus agalactiae*	***pscB***	GAGTCTGCCACAGCTCCTACTGAAACAGTTCAAACACAACCGAGAACTGAAATAAAGCCTTCTAATCTTA
SaGAL4	*Streptococcus agalactiae*	***pscB***	TTGCAGTAGTAACGTCAGTGGCTAACAATTCATCTATCCAAGTTATGGAATCAAACTATGCTGGAAATAT
SaGAL5	*Streptococcus agalactiae*	***sip***	TAAAGTAGCACCGGTAAGAACTGTAGCAGCCCCTAGAGTGGCAAGTGTTAAAGTAGTCACTCCTAAAGTA
SaGAL6	*Streptococcus agalactiae*	***sip***	TCTCTCAATACAATTTCGGAAGGTATGACACCAGAAGCAGCAACAACGATTGTTTCGCCAATGAAGACAT
SaGAL7	*Streptococcus agalactiae*	***sip***	TAAGTTACAAGCGACTGAAGTTAAGAGCGTTCCGGTAGCACAAAAAGCTCCAACAGCAACACCGGTAGCA
SaGAL8	*Streptococcus agalactiae*	***sip***	TTCTCTCAATACAATTTCGGAAGGTATGACACCAGAAGCAGCAACAACGATTGTTTCGCCAATGAAGACA
SPne1	*Streptococcus pneumoniae*	***ply***	TGATATTTCTGTAACAGCTACCAACGACAGTCGCCTCTATCCTGGAGCACTTCTCGTAGTGGATGAGACC
SPne2	*Streptococcus pneumoniae*	***ply***	TCCAGGAGATGTGTTTCAAGATACTGTAACGGTAGAGGATTTAAAACAGAGAGGAATTTCTGCAGAGCGT
SPne3	*Streptococcus pneumoniae*	***dexB***	TCGAGCTGACTTTGAATTGCTTGATACGGCTGATAAGGTCTTTGCTTATATACGTAAGGATGGCGACCGT
SPne4	*Streptococcus pneumoniae*	***dexB***	ACAATGCCCGTACCCCTATGCAATGGGACGAGAGCAAAAACGCTGGTTTCTCAACAGGTCAACCTTGGTT
SPne5	*Streptococcus pneumoniae*	***dexB***	GAGAGCAAAAACGCTGGTTTCTCAACAGGTCAACCTTGGTTGGCGGTTAATCCAAATTACGAGATGATCA
SPne6	*Streptococcus pneumoniae*	***dexB/ intergenic***	TTTAGCTGCTTGCGGCCAATCAGGTTCAGATACAAAAACTTACTCATCAACCTTTAGTGGAAATCCAACT
SPne7	*Streptococcus pneumoniae*	**capN-like gene**	CGCCTCTAGCTAATTACAAGGGTAGCCTTTATAATCTACCTTTCAATATGAATACTTTCTATGCTATGTG
SPne8	*Streptococcus pneumoniae*	**aliB-like gene**	TGATTCAAAACTCGATTAAGGGCTTGAATGATTATATTATAGGAGCGGATTCTGACTTTTCTAAGGTTGG
SPne9	*Streptococcus pneumoniae*	***sulB***	CTAACTACCGGACGGATCAACCGCATTTTGGCTTGGAACGAATGGTGGAACTGTTAGCTTTGCGTGGCAA
SPne10	*Streptococcus pneumoniae*	***sulB***	TTTAGCTCGCCCTATCTCATTCATTACACAGACCAGATTAGCATCAATGGGGAATCGATCTCAGAAGCGA
SPne11	*Streptococcus pneumoniae*	***sulB***	ACAACCGAGTTTGAGATTATCACAGCCCTGGCCTATGACTACTTTGCCTCAGAGCAAGTAGATGTGGCCA
SPne12	*Streptococcus pneumoniae*	***sulB***	AAAGATGCGCCGAGACTTGCCTACGGGACAGATTATCAGGTTCGTCATCAAGAAAGTGTGGTGACAGGGG
Strep 1	*Streptococcus* spp.	***16S rRNA***	GTTAGTTATTTAAAAGGTGCAATTGCATCACTATGAGATGGACCTGCGT
Strep 2	*Streptococcus* spp.	***16S rRNA***	AGCTCTGTTGTAAGAGAAGAACGAGTGTGAGAGTGGAAAGTTCACACT

StAU1	*Staphylococcus aureus*	**NWMN_ 2357**	GATCAATCTTTGTCGGTACACGATATTCTTCACGACTAAATAAACGCTCATTCGCGATTTTATAAATGAA
StAU2	*Staphylococcus aureus*	**NWMN_ 2357**	TGTTGATAACAATGTTGTATTATCTACTGAAATCTCATTACGTTGCATCGGAAACATTGTGTTCTGTATG
StAU3	*Staphylococcus aureus*	**NWMN_ 2357**	TAAAAGCCGTCTTGATAATCTTTAGTAGTACCGAAGCTGGTCATACGAGAGTTATATTTTCCAGCCAAAA
StAU4	*Staphylococcus aureus*	**NWMN_ 1707/ 1708**	TGTTTTGAATTATAAAAACTAAAGTAAAAATGTTGGATGTGAAAGATTGTTGATGAGAATTTTGAGATGT
StAU5	*Staphylococcus aureus*	***SplB***	TTACCGATGCAACTAAGGAACCATACAATTCAGTGGTAGCATTTGTGGGTGGTACTGGTGTAGTTGTTGGT
StAU6	*Staphylococcus aureus*	***SplF***	AAATTTAATATAGCATCAGAAGCTAAAGAAAATGAACCTATATCAGTCATTGGTTATCCAAATCCTAATGG
StAU7	*Staphylococcus aureus*	***SplC***	CGTTGCAACATATCTCGAATTGTAAAGGAGCTTGAAAATGAATAAAAATATAGTCATTAAAAGCATGGCAG
StAU8	*Staphylococcus aureus*	***SplC***	TGCAGTCGTTGAAGAGACACAACAAATAGCAAATGCAGAGAAGAATGTTACGCAAGTTAAAGATACAAATAT
StAUA1	*Staphylococcus aureus*	***spa***	TGGTTTTATCCAAAGCCTTAAAGATGATCCAAGCCAAAGTGCTAACGTTTTAGGTGAAGCTCAAAAACTT
StAUA2	*Staphylococcus aureus*	***spa***	AGCCAAAGTGCTAACGTTTTAGGTGAAGCTCAAAAACTTAATGACTCTCAAGCTCCAAAAGCTGATGCGC
StAUA3	*Staphylococcus aureus*	***spa***	TCAACAAAGAACAACAAAATGCTTTCTATGAAATCTTACATTTACCTAACTTAAACGAAGAACAACGCAA
StAUA4	*Staphylococcus aureus*	***spa***	AGAACAACAAAATGCTTTCTATGAAATTTTACATTTACCTAACTTAACTGAAGAACAACGTAACGGCTTC
StAUB1	*Staphylococcus aureus*	***spa***	GATCAACGTAATGGTTTTATCCAAAGCCTTAAAGATGATCCAAGCCAAAGTGCTAACGTTTTAGGTGAAG
StAUB2	*Staphylococcus aureus*	***spa***	AAAGTGCTAACGTTTTAGGTGAAGCTCAAAAACTTAATGACTCTCAAGCTCCAAAAGCTGATGCGCAACA
StAUB3	*Staphylococcus aureus*	***spa***	GAATCTCAAGCACCGAAAGCTGATAACAATTTCAACAAAGAACAACAAAATGCTTTCTATGAAATCTTGA
StAUB4	*Staphylococcus aureus*	***spa***	CGGCTTCATCCAAAGCCTTAAAGACGATCCTTCAGTGAGCAAAGAAATTTTAGCAGAAGCTAAAAAGCTA

<u>Supplementary Material S2, Table S3: Additional Microarray Oligonucleotide Probes used in Study</u>

Probe	Organism	Gene	Sequence
TB_ESAT6_1	*Mycobacterium tuberculosis*	**ESAT6**	TATCGAGGCCGCGGCAAGCGCAATCCAGGGAAATGTCACGTCCATTCATTCCCTCCTTGACGAGGGGAAG
TB_ESAT6_2	*Mycobacterium tuberculosis*	**ESAT6**	CGCAGCGGCCTGGGGCGGTAGCGGTTCGGAGGCGTACCAGGGTGTCCAGCAAAAATGGGACGCCACGGCT
MTB1	*Mycobacterium tuberculosis*	**IS6110**	GTCAGCACGATTCGGAGTGGGCAGCGATCAGTGAGGTCGCCCGTCTACTTGGTGTTGGCTGCGCGGAGAC
MTB2	*Mycobacterium tuberculosis*	**IS6110**	AAGCGCTTGCGGCGGGACAACGCCGAATTGCGAAGGGCGAACGCGATTTTAAAGACCGCGTCGGCTTTCT
MTB3	*Mycobacterium tuberculosis*	**16S rRNA**	AGTGGCGAACGGGTGAGTAACACGTGGGTGATCTGCCCTGCACTTCGGGATAAGCCTGGGAAACTGGGTC
MTB4	*Mycobacterium tuberculosis*	**16S rRNA**	ACCGGATAGGACCACGGGATGCATGTCTTGTGGTGGAAAGCGCTTTAGCGGTGTGGGATGAGCCCGCGGCC
G6PD1	*Homo sapiens*	**G6PD**	CCGTAGGCAGCCTCTCTGCTATAAGAAAAGCAGACGCAGCAGCTGGGACCCCTCCCAACCTCAATGCCCT
PGK1	*Homo sapiens*	**PGK**	AAAGTCAAGGCTTATAACAAAAAAGCCCCAGCCCATTCCTCCCATTCAAGATTCCCACTCCCCAGAGGTG
B2M1	*Homo sapiens*	**B2M**	AGGAGGGCTGGCAACTTAGAGGTGGGGAGCAGAGAATTCTCTTATCCAACATCAACATCTTGGTCAGAT
APRR5A	*Arabidopsis thaliana*	**APRR5**	AGGAGCCATTGCAGATGTATAAGCTGTGTTGGGATCTCTGAACTGTATATTGTTGATAGGGGTTGGCGTA
