# Supplementary material for: Development and Assessment of a Diagnostic DNA Oligonucleotide Microarray for Detection and Typing of Meningitis-Associated Bacterial Species
Source: High Throughput. 2018 Oct 16;7(4):32. doi: 10.3390/ht7040032 (PMC6306750; doi:10.3390/ht7040032)
Supplement: Supplementary file 1 [file high-throughput-07-00032-s001.zip › Supplementary Material S3.pdf]

Supplementary Material S3, Table S4: List of PCR Primers for Target Amplification used in Study

Primer	Target Probe	Organism	Gene	Sequence
PrimerHi_HAI1_R	HAI1	*Haemophilus influenzae*	***nucA***	TGAAAATTACCTGCGTTCAT
PrimerHi_HAI1_F	HAI1	*Haemophilus influenzae*	***nucA***	AGCTGTGGAATTAAGTATTTT
PrimerHi_HAI2_R	HAI2	*Haemophilus influenzae*	***nucA***	CATCATTTCCGTATAAATAATG
PrimerHi_HAI2_F	HAI2	*Haemophilus influenzae*	***nucA***	CCTGATAAAAGTTCAATTTTG
PrimerHi_HAI3_R	HAI3	*Haemophilus influenzae*	***nucA***	ACGAACCTTCCATTTTATAG
PrimerHi_HAI3_F	HAI3	*Haemophilus influenzae*	***nucA***	AATAAAGCAGGATCTAATCC
PrimerHi_HAI4_R	HAI4	*Haemophilus influenzae*	***nucA***	ATTTGTTCCGACAAGATAAC
PrimerHi_HAI4_F	HAI4	*Haemophilus influenzae*	***nucA***	ACCTTTAACGATGCTTATAC
PrimerHi_HAI5_7_R	HAI5	*Haemophilus influenzae*	***cpdB***	TTCCGCACTTATAACTTTGA
PrimerHi_HAI5_7_F	HAI5	*Haemophilus influenzae*	***cpdB***	TTCTCACTGGTTGCTTTAAT
PrimerHi_HAI6_R	HAI6	*Haemophilus influenzae*	***cpdB***	AAAGCAGAATTTTTGATTGC
PrimerHi_HAI6_F	HAI6	*Haemophilus influenzae*	***cpdB***	TTCATTGGGTATTGTGCTTT
LiMo2_3_R	LiMo2	*Listeria monocytogenes*	***dltA***	ACCCAAGTGAAAGTAACTCGAGAG AT
LiMo2_3_F	LiMo2	*Listeria monocytogenes*	***dltA***	CCTGTGCGATAGGCTTGATAACCT
LiMo4_6_R	LiMo4	*Listeria monocytogenes*	***hlyAB***	TTGCCGAAAAATCTGGAAGGTCTTGT
LiMo4_6_F	LiMo4	*Listeria monocytogenes*	***hlyAB***	CAACCAGATGTTCTCCCTGTAAAACG
Neiss1_2_R	Neiss1/2	*Neisseria spp.*	**16S rRNA**	CGCCCAGTAATTCCG ATTAACGCT
Neiss1_2_F	Neiss1/2	*Neisseria spp.*	**16S rRNA**	CCGCATACGCTTTGAGAAGGAAAG C
NsMA4_R	NsMA4	*Neisseria spp.*	**type IV PRP**	ACGCTGTTCATCCATACCGACAAAG
NsMA4_F	NsMA4	*Neisseria spp.*	**type IV PRP**	CCCCTGGACGATAATCAGACCAT
NsMA7_8_R	NsMA7	*Neisseria meningitidis* A	***sacB***	ATGGTGCAAGCTGGTTTCAATAGCA
NsMA7_8_F	NsMA7	*Neisseria meningitidis* A	***sacB***	GGGTCAACTCAGAAGATAAGAATTGG
NsMa9_R	NsMa9	*Neisseria meningitidis* A	***sacB***	GTGCTGAGTTTCATGTCAACATGAGC
NsMa9_F	NsMa9	*Neisseria meningitidis* A	***sacB***	AGCTCAATCAAATATTTCTTCAACCT
NsMC1_ R	NsMCD1	*Neisseria meningitidis* C/D	***SiaD/SynC***	TGTGCAGGTTGGATAGTTTTGAGAAG
NsMC1_ F	NsMCD1	*Neisseria meningitidis* C/D	***SiaD/SynC***	ACCACACAAAATATGAAAATGCCGAA

NsMC2_ R	NsMCD2	*Neisseria meningitidis* C/D	***SiaD/SynC***	CTGCTTAACTTTATTAAGGGCATTGT
NsMC2_ F	NsMCD2	*Neisseria meningitidis* C/D	***SiaD/SynC***	AGCCCAACAAGAGAACACCGC
NsMC4_5_ R	NsMCD4/5	*Neisseria meningitidis* C/D	***SiaD/SynC***	ATCACTACTAGACGGGGATTAATTGT
NsMC4_5_ F	NsMCD4/5	*Neisseria meningitidis* C/D	***SiaD/SynC***	CAACCTATGCCCCACTCTTAGAATCA
NsMW1_ F	NsMW1-4	*Neisseria meningitidis* W	***synG***	AGGTGAATCTTCCGAGCAGGAAATT
NsMW1_ R	NsMW1-4	*Neisseria meningitidis* W	***synG***	AAGCTGCGCGGAAGAATAGTGA
NsMX1_4_ F	NsMX1-4	*Neisseria meningitidis* X	***xcbA/B***	GCAAAGCAAAAAACTTCGTACACTGA
NsMX1_4_ R	NsMX1-4	*Neisseria meningitidis* X	***xcbA/B***	GGGCAAACAATACCAATAAACGGTTG
NsMX2_ F	NsMX2	*Neisseria meningitidis* X	***xcbB***	ACAAATGCGACATCCAGCCTGA
NsMX2_ R	NsMX2	*Neisseria meningitidis* X	***xcbB***	AGTATTGCCGCGTAGATGATTAACAA
NsMX5_ F	NsMX5	*Neisseria meningitidis* X	***xcbB***	ACGCAGCTTGGTCAAAAACGTCTA
NsMX5_ R	NsMX5	*Neisseria meningitidis* X	***xcbB***	GCGCCACCTTTACTGCCTCCAT
NsMY1_5_ F	NsMY1-5	*Neisseria meningitidis* Y	***synF***	GGTGAATCTTCCGAGCAGGAAATTTA
NsMY1_5_ R	NsMY1-5	*Neisseria meningitidis* Y	***synF***	AGCTGCGCGGAAGAATAGTGAAA
SaGAL1_2_3_ F	SaGAL1-3	*Streptococcus agalactiae*	***pcsB***	ATCGTGTTGTTGCTATTCGTGAGGT
SaGAL1_2_3_ R	SaGAL1-3	*Streptococcus agalactiae*	***pcsB***	GCAACAGTAGTTGCCGAAGAAGTAGC
SaGAL4__ F	SaGAL4	*Streptococcus agalactiae*	***pcsB***	CGTGCAGCTGGTTATTCTGTAGGAAC
SaGAL4__ R	SaGAL4	*Streptococcus agalactiae*	***pcsB***	AGCACTAGGGTTAAATGAACCACGG
SaGAL5_7_ F	SaGAL5&7	*Streptococcus agalactiae*	***sip***	AGCTTCTGTTGCCGCTGAAACAC
SaGAL5_7_ R	SaGAL5&7	*Streptococcus agalactiae*	***sip***	GGAGCCTTGCATTTTCAGGATGTG
SaGAL6_8_ F	SaGAL6&8	*Streptococcus agalactiae*	***sip***	ACCAATCAAGTTTCTGTTGCAGACCA
SaGAL6_8_ R	SaGAL6&8	*Streptococcus agalactiae*	***sip***	GGAGCTGGTGATACCTGTTCATTAGC
Strep1_3 F	Strep1-3	*Streptococcus* spp.	***16S rRNA***	CCATTGCCGAAGATTCCCTACT
Strep1_3 R	Strep1-3	*Streptococcus* spp.	***16S rRNA***	TGGAAACGATAGCTAATACCGCATAA
SPne1_ F	SPne1	*Streptococcus pneumoniae*	***ply***	CAGCTACCCGATGAGTTTGTTGTTAT
SPne1_ R	SPne1	*Streptococcus pneumoniae*	***ply***	GCTGGGGTCTTCCACTTGGAGA
SPne10_11_ F	SPne10-11	*Streptococcus pneumoniae*	***sulB***	CAAGGGCTCGACTATTGCTTTTTGA
SPne10_11_ R	SPne10-11	*Streptococcus pneumoniae*	***sulB***	AAGTCCACCCATGCCAACTTCC
SPne12_ F	SPne12	*Streptococcus pneumoniae*	***sulB***	CTCCAGAAGCCTTGGCTGTGATTGA

SPne12_ R	SPne12	*Streptococcus pneumoniae*	***sulB***	AGGCTAGTCTGGAAGCGACCT
SPne2_ F	SPne2	*Streptococcus pneumoniae*	***ply***	ACTCTGTCCATTCAGGCGAAAAGC
SPne2_ R	SPne2	*Streptococcus pneumoniae*	***ply***	AGCCTCTACTTCATCACTCTTACTCG
SPne3_4_5_ F	SPne3-5	*Streptococcus pneumoniae*	***dexB***	CCGATTGAAGAAATCATGGACAGTAT
SPne3_4_5_ R	SPne3-5	*Streptococcus pneumoniae*	***dexB***	GCCGCAGTGTTTTCAATCAAGACAGA
SPne6_ F	SPne6	*Streptococcus pneumoniae*	***dexB***	CGATTGCGGCTATTTTTGGAACCAT
SPne6_ R	SPne6	*Streptococcus pneumoniae*	***dexB***	GTTCATCGCGCCAGATATAGTAGTCT
SPne7_ F	SPne7	*Streptococcus pneumoniae*	***cap***	CCGATGGAAGAAATCATGGACAGTAT
SPne7_ R	SPne7	*Streptococcus pneumoniae*	***cap***	CCAGCGCTTCTTGAACGTTGATTTC
SPne9_ F	SPne9	*Streptococcus pneumoniae*	***sulB***	GAAAGAAATTGAAAACAATCAATGGA
SPne9_ R	SPne9	*Streptococcus pneumoniae*	***sulB***	GTAATGAATGAGATAGGGCGAGC
StAU1_2_3-F	StAU1-3	*Staphylococcus aureus*	**NWMN_2357**	GCGCCATAACTCATACCAGATTGTCC
StAU1_2_3-R	StAU1-3	*Staphylococcus aureus*	**NWMN_2357**	ATTTTCTGGGGATAATGAAGGGAAAC
StAU11_femA-F	StAU11	*Staphylococcus aureus*	***femA***	CAAGAACATGGCAATGAATTACCCAT
StAU11_femA-R	StAU11	*Staphylococcus aureus*	***femA***	AACTTAACTACGCCAGCATCTTCAG
StAU3_Spa-F	StAU3	*Staphylococcus aureus*	***Spa***	GGCAAAGAAGATGGCAACAAACCTG
StAU3_Spa-R	StAU3	*Staphylococcus aureus*	***Spa***	CCGTTGCCGTCTTCTTTACCAGG
StAU4-F	StAU4	*Staphylococcus aureus*	**NWMN_1707/1708**	TGCTCCTTTTTTTATATTGTAAACG
StAU4-R	StAU4	*Staphylococcus aureus*	**NWMN_1707/1708**	ACTGACATGTTGATGCATGTCTT
StAU5-F	StAU5	*Staphylococcus aureus*	***SplB***	CACGCTCTTCAACTTGAATGACTGAT
StAU5-R	StAU5	*Staphylococcus aureus*	***SplB***	CATCAAGAGTTTAGCAGCATTAACAA
StAU6-F	StAU6	*Staphylococcus aureus*	***SplF***	ACTTATCACATGAAAGTCGGTGATGA
StAU6-R	StAU6	*Staphylococcus aureus*	***SplF***	ACAACCGCATCAGATGTCACTATAT
StAU7_8-F	StAU7&8	*Staphylococcus aureus*	***SplC***	ATGGCGTCTACTTTACACCAGAAAT
StAU7_8-R	StAU7&8	*Staphylococcus aureus*	***SplC***	CTGTCGCATCTTTAAATGAAACGAC
StAU9_mecA-F	StAU9	*Staphylococcus aureus*	***mecA***	CAAACTACGGTAACATTGATCGCAAC
StAU9_mecA-R	StAU9	*Staphylococcus aureus*	***mecA***	TTGGAACGATGCCTATCTCATATGC
StAUA1_2_B1_2-F	StAUA1&2/B1&2	*Staphylococcus aureus*	***spa***	CACGATGAAGCTCAACAAAATGCTTT
StAUA1_2_B1_2-R	StAUA1&2/B1&2	*Staphylococcus aureus*	***spa***	AGGCATGTTCAAGATTTCATAGAAGG

StAUA1_2B1_2_Spa-F	StAUA1&2/B1&2	*Staphylococcus aureus*	***spa***	GTGGCGTAACACCTGCTGCAAA
StAUA1_2B1_2_Spa-R	StAUA1&2/B1&2	*Staphylococcus aureus*	***spa***	ACCAGGCTTGTTGTTGTCTTCTTTGC
StAUA10_femBb-F	StAUA10	*Staphylococcus aureus*	***spa***	CTGAGTATGATACATCGAGCCAAG
StAUA10_femBb-R	StAUA10	*Staphylococcus aureus*	***spa***	ATACAAATCCAGCACGCTCTTC
StAUA10_femB-F	StAUA10	*Staphylococcus aureus*	***spa***	ACTGAGTATGATACATCGAGCCAAG
StAUA10_femB-R	StAUA10	*Staphylococcus aureus*	***spa***	ACAAATCCAGCACGCTCTTCAGT
StAUA3_4_B4b-F	StAUA3	*Staphylococcus aureus*	***spa***	GTTGTCTTCCTCTTTTGGTGCTT
StAUA3_4_B4b-R	StAUA3	*Staphylococcus aureus*	***spa***	AAACGAAGAACAACGCAATGGT
StAUA3_4_B4-F	StAUA3	*Staphylococcus aureus*	***spa***	CGAAGAACAACGCAATGGTTTCATCC
StAUA3_4_B4-R	StAUA3	*Staphylococcus aureus*	***spa***	GTTGTCTTCCTCTTTTGGTGCTTGAG
TB_ESAT6_1-F	TB_ESAT6	*Mycobacterium tuberculosis*	***ESAT6***	ATGACAGAGCAGCAGTGGAATTT
TB_ESAT6_1-R	TB_ESAT6	*Mycobacterium tuberculosis*	***ESAT6***	ATGCGAACATCCCAGTGACGTTG
MTB1_2b-F	MTB1&2	*Mycobacterium tuberculosis*	**IS6110**	GAGGAGGTACCCGCCGGAGCTG
MTB1_2b-R	MTB1&2	*Mycobacterium tuberculosis*	**IS6110**	TTAGCGTGCTGGCCGGTCGA
MTB1_2-F	MTB1&2	*Mycobacterium tuberculosis*	**IS6110**	ATGTCAGGTGGTTCATCGAGGA
MTB1_2-R	MTB1&2	*Mycobacterium tuberculosis*	**IS6110**	TTAGCGTGCTGGCCGGTC
MTB3_4-F	MTB3&4	*Mycobacterium tuberculosis*	**16S rRNA**	GTTCCGCTTCGTTGAAGAGATGATCC
MTB3_4-R	MTB3&4	*Mycobacterium tuberculosis*	**16S rRNA**	CGCCTAACAGCATACCCGTTCG
B2M1-F	B2M1	*Homo sapiens*	**B2M**	CTACTTTGAGTGCTGTCTCCATGTT
B2M1-R	B2M1	*Homo sapiens*	**B2M**	AGCTTTGAGTGCAAGAGATTGAAGAG
G6PD-F	G6PD1	*Homo sapiens*	**G6PD**	GTCCCACCAACTCTGCACTCCA
G6PD-R	G6PD1	*Homo sapiens*	**G6PD**	TTTTTTGGCTGTTTGCGGATTTAATG
PGK-F	PGK1	*Homo sapiens*	**PGK**	CAGCAGCAGTGGAGAGATGGG
PGK-R	PGK1	*Homo sapiens*	**PGK**	GGAACAGAGCCTTCCTCCATGGTA
APRR5A-F	APRR5A	*Arabidopsis thaliana*	**APRR5**	AGGAGCCATTGCAGATGTATAAGC
APRR5A-R	APRR5A	*Arabidopsis thaliana*	**APRR5**	AAGCCAACAGCAATGCACCATCAG
