# Supplementary material for: Development and Assessment of a Diagnostic DNA Oligonucleotide Microarray for Detection and Typing of Meningitis-Associated Bacterial Species
Source: High Throughput. 2018 Oct 16;7(4):32. doi: 10.3390/ht7040032 (PMC6306750; doi:10.3390/ht7040032)
Supplement: Supplementary file 1 [file high-throughput-07-00032-s001.zip › Supplementary Material S4.pdf]

Supplementary Material 4, Table S5: List of PCR Primers for Real-Time PCR used in Study

Primer/probe (5′ fluorophor; 3′ quencher)	Target	Organism	Sequence (5′→3′)
Forward	*ctr*A	*N.meningitidis*	GCTGCGGTAGGTGGTTCAA
Reverse			TTGTCGCGGATTTGCAACTA
Probe (5′FAM; 3′TAMRA)			CATTGCCACGTGTCAGCTGCACAT
Forward	*fem*A	*S.epidermidis*	CAACTCGATGCAAATCAGCAA
Reverse			GAACCGCATAGCTCCCTGC
Probe (5′CY5; 3′BHQ)			TGAAGCTAAAAACTTAAAACAAGAACATGGCAATGA
Forward	*ply*	*Str.pneumoniae*	TGCAGAGCGTCCTTTGGTCTAT
Reverse			CTCTTACTCGTGGTTTCCAACTTGA
Probe (5′CY5; 3′BHQ)			TGGCGCCCATAAGCAACACTCGAA
Forward	*hel*	*H. influenzae*	CCGGGTGCGGTAGAATTTAATAA
Reverse			CTGATTTTTCAGTGCTGTCTTTGC
Probe (5′CY5; 3′BHQ)			ACAGCCACAACGGTAAAGTGTTCTACG
Forward	*sip*	*Str.agalactiae* (group B streptococcus)	ATCCTGAGACAACACTGACA
Reverse			TTGCTGGTGTTTCTATTTTCA
Probe (5′FAM; 3′BHQ)			ATCAGAAGAGTCATACTGCCACTTC
Forward	*fem*B	*S.aureus*	GACATTTGATAGTCAACGTAAACGTAATATT
Reverse			GCTCTTCAGTTTCACGATATAAATCTAAGA
Probe (5′HEX; 3′TAMRA)			TCATCACGTTCAAGGAATCTGACTTTAACACCATAGT
Forward	*hly*	*L.monocytogenes*	CATGGCACCACCAGCATCT
Reverse			ATCCGCGTGTTTCTTTTCGA
Probe (5′FAM; 3′TAMRA)			CGCCTGCAAGTCCTAAGACGCCA
