# Supplementary material for: Development and Assessment of a Diagnostic DNA Oligonucleotide Microarray for Detection and Typing of Meningitis-Associated Bacterial Species
Source: High Throughput. 2018 Oct 16;7(4):32. doi: 10.3390/ht7040032 (PMC6306750; doi:10.3390/ht7040032)
Supplement: Supplementary file 1 [file high-throughput-07-00032-s001.zip › Supplementary Material S8.pdf]

Supplementary Material S8; Table S14

Patient	Sample	Culture Identification	Supernatant Sample	Total Nucleic Acid in Supernatant Fraction (ng)	Pellet Sample	Total Nucleic Acid in Pellet Fraction (ng)
**1**	Cerebrospinal Fluid	Negative	CSF1S	280	CSF1P	2440
**2**	Cerebrospinal Fluid	Negative	CSF2S	200	CSF2P	320
**3**	Cerebrospinal Fluid	Negative	CSF3S	300	CSF3P	20
**4**	Cerebrospinal Fluid	Negative	CSF4S	300	CSF4P	300
**5**	Cerebrospinal Fluid	Negative	CSF5S	140	CSF5P	0
**6**	Cerebrospinal Fluid	Negative	CSF6S	140	CSF6P	640
**7**	Cerebrospinal Fluid	Negative	CSF7S	540	CSF7P	1600
