# Supplementary figures and images for: Development and Assessment of a Diagnostic DNA Oligonucleotide Microarray for Detection and Typing of Meningitis-Associated Bacterial Species
Source: High Throughput. 2018 Oct 16;7(4):32. doi: 10.3390/ht7040032 (PMC6306750; doi:10.3390/ht7040032)

# Supplementary Material S10

4

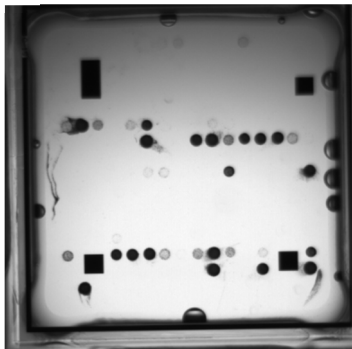

11

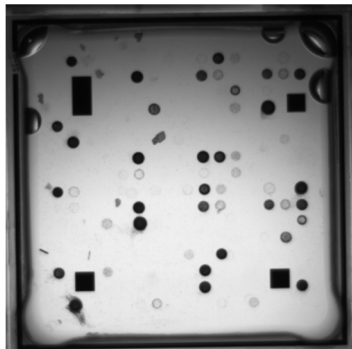

13

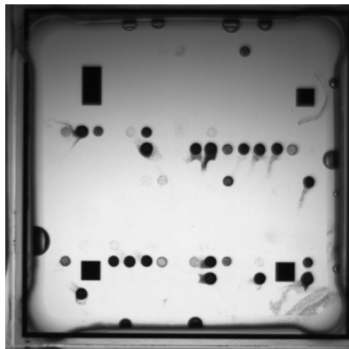

14

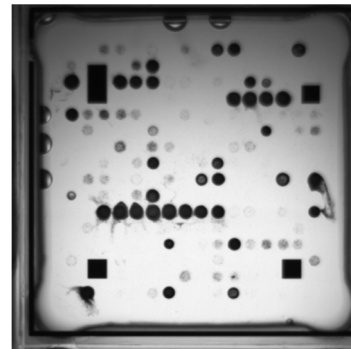

21

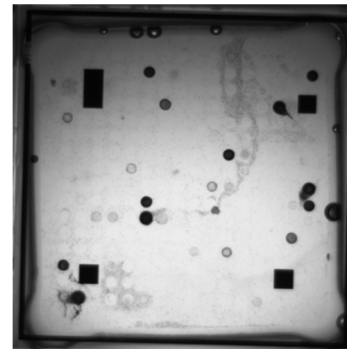

24

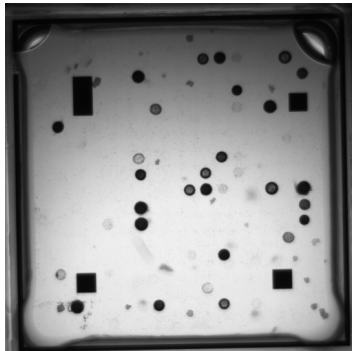

29

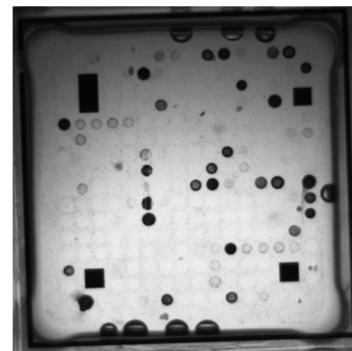

34

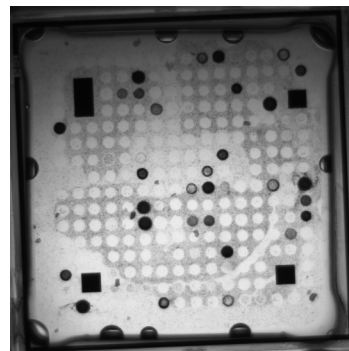

51

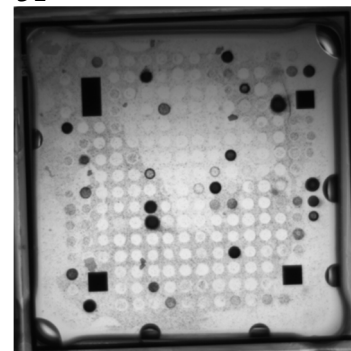

54

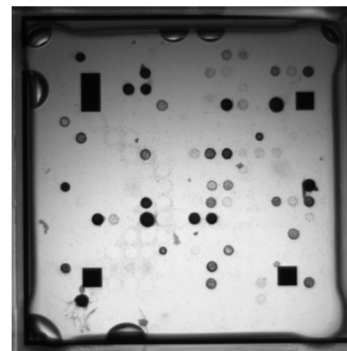

61

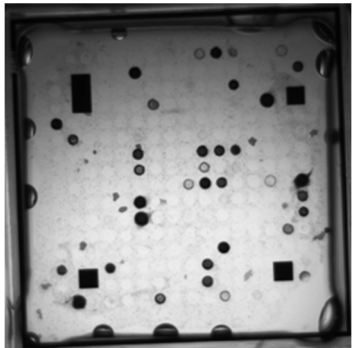

63

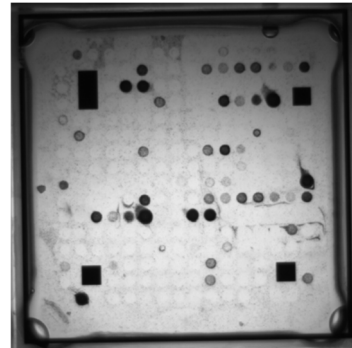

70

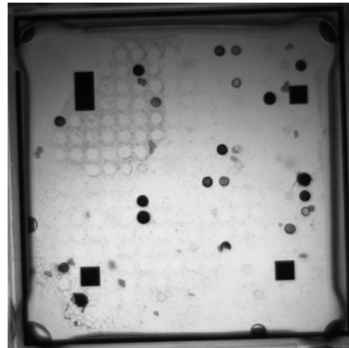

72

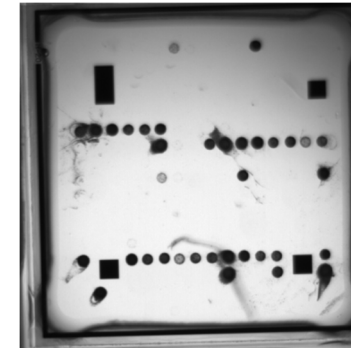

76

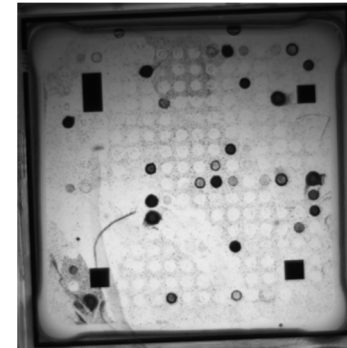

90

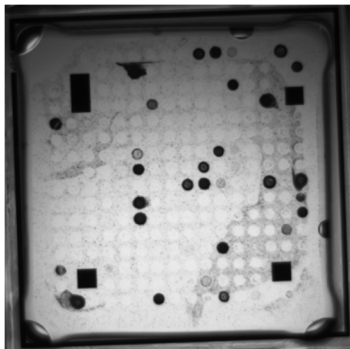

95

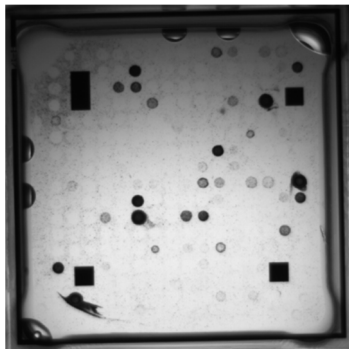

127

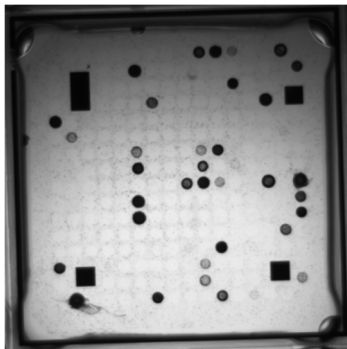

145

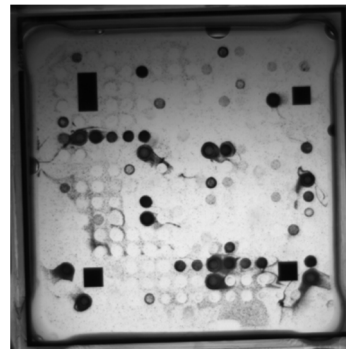

147

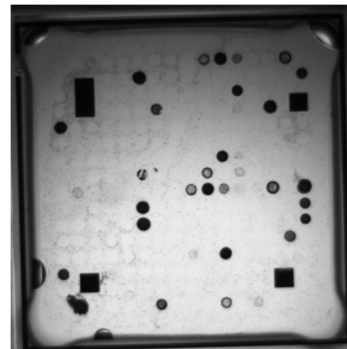

no template control

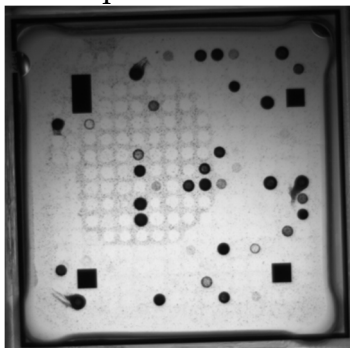

Supplement: Supplementary file 1 [file high-throughput-07-00032-s001.zip › Supplementary Material S10.pdf]
